# Effect of Berberine Phytosome on reproductive, dermatologic, and metabolic characteristics in women with polycystic ovary syndrome: a controlled, randomized, multi-centric, open-label clinical trial

**DOI:** 10.3389/fphar.2023.1269605

**Published:** 2023-11-21

**Authors:** Francesco Di Pierro, Ruqqia Sultana, Amna Zia Eusaph, Saida Abrar, Mahroo Bugti, Fauzia Afridi, Umer Farooq, Somia Iqtadar, Fareeha Ghauri, Syeda Makhduma, Shazia Nourin, Ayesha Kanwal, Aasiya Bano, Ali Akbar Bugti, Shah Mureed, Ayesha Ghazal, Romana Irshad, Martino Recchia, Alexander Bertuccioli, Pietro Putignano, Antonella Riva, Luigina Guasti, Nicola Zerbinati, Amjad Khan

**Affiliations:** ^1^ Scientific and Research Department, Velleja Research, Milan, Italy; ^2^ Department of Medicine and Surgery, University of Insubria, Varese, Italy; ^3^ Department of Gynaecology and Obstetrics, Ayub Teaching Hospital, Abbottabad, Pakistan; ^4^ Department of Gynaecology and Obstetrics, Sir Ganga Ram Hospital, Fatima Jinnah Women University, Rawalpindi, Pakistan; ^5^ Department of Gynaecology and Obstetrics, Lady Reading Hospital, Peshawar, Pakistan; ^6^ Department of Gynaecology and Obstetrics, Bolan Medical Complex Hospital, Quetta, Pakistan; ^7^ Department of Gynaecology and Obstetrics, Khyber Teaching Hospital, Peshawar, Pakistan; ^8^ Department of Community Medicine, Ayub Teaching Hospital, Abbottabad, Pakistan; ^9^ Department of Medicine, King Edward Medical University, Lahore, Pakistan; ^10^ PEOC, Department of Health, Quetta, Balochistan, Pakistan; ^11^ Department of General Surgery, Bolan Medical Complex Hospital, Quetta, Pakistan; ^12^ Department of Paediatrics, Bolan Medical Complex Hospital, Quetta, Pakistan; ^13^ Department of Microbiology, University of Health Sciences, Lahore, Pakistan; ^14^ Department of Pathology, Ayub Teaching Hospital, Abbottabad, Pakistan; ^15^ Unit of Clinical Epidemiology and Biostatistics, Mario Negri Institute Alumni Association (MNIAA), Milan, Italy; ^16^ Department of Biomolecular Sciences, University of Urbino Carlo Bo, Urbino, Italy; ^17^ SP Diabetic Outpatient Clinic, Monza, Italy; ^18^ Indena S.p.A, Milan, Italy; ^19^ Nuffield Division of Clinical Laboratory Sciences, Radcliffe Department of Medicine, University of Oxford, Oxford, United Kingdom; ^20^ Department of Biochemistry, Liaquat University of Medical and Health Sciences, Jamshoro, Pakistan

**Keywords:** berbevis, phytosome, insulin, glucose, cholesterol, testosterone, PCOS

## Abstract

**Background:** Berberine is a poorly absorbed natural alkaloid widely used as nutraceutical to counteract diarrhoea and to lower cholesterol and hyperglycaemia. It has also been reported to reduce signs and symptoms of polycystic ovary syndrome (PCOS).

**Objective:** To explore, through a multi-centric, randomized, controlled and prospective study, the possible role played by a form berberine that is more easily absorbed (Berberine Phytosome^®^, BP) in 130 Pakistani women with a diagnosis of PCOS and fertility problems due to menstrual and ovary abnormalities.

**Results:** Ninety days of supplementation with BP, administered at 550 mg x2/*die*, determined (i) resumption of regular menstruation in about 70% of women (*versus* 16% in the control group; *p* < 0.0001), (ii) normalization of the ovaries anatomy in more than 60% of women (*versus* 13% in the control group; *p* < 0.0001), (iii) acne improvement in 50% of women (*versus* 16% in the control group; *p* = 0.0409) and (iv) hirsutism reduction in 14% of women (*versus* 0% in the control group; *p* = 0.0152). The metabolic and the hormonal profiles of the women in the two groups did not significantly differentiate at the end of the study. BP was well-tolerated and no specific side-effects were registered. Respectively after one, two and 8 years of trying, three women supplemented with BP became and are currently pregnant.

**Conclusion:** Our study showed the positive effects of BP supplementation in women with PCOS and confirmed the high safety profile of this nutraceutical.

**Clinical Trial Registration:** https://clinicaltrials.gov/, identifier NCT05480670

## Introduction

Polycystic ovary syndrome (PCOS) is the most common endocrine disease of women of reproductive age (5%–15% depending on the diagnostic criteria applied), the main cause of anovulatory infertility, a likely contributor to the early onset of type-2 diabetes as well as a factor in the occurrence of anxiety and depression (Hamilton-Fairley and Anovulation, 2003; Lizneva et al., 2016; History of discovery of polycystic ovary syndr ome, 2017; Santoro, 2018; Kakoly et al., 2019; Joham et al., 2022). In 2018, the so-called Rotterdam Criteria, ratified that, to make a correct diagnosis of PCOS, two out of three features must be evident in adults: menstrual disturbance, hyperandrogenism and multi-follicular ovarian morphology (Dokras et al., 2018). Hyperandrogenism is likely the key feature of PCOS (Ruth et al., 2020). The main source of this androgen excess is the ovarian androgen hypersecretion (Legro et al., 1998). In turn, ovarian hyperandrogenism is due to gonadotropin releasing hormone (GnRH) causing luteinizing hormone (LH) secretion, which further stimulates the ovarian theca cells to produce androgen (Stener-Victorin et al., 2010). This peculiar hormonal mix inhibits follicular maturation, causing the typical excess of small antral follicles, ovulatory disturbance and an increase of the granulosa-derived anti-mullerian hormone, two to three times above normal levels (Moolhuijsen et al., 2020). The main clinical features of PCOS are reproductive (menstrual irregularity, anovulation, infertility and pregnancy complications), dermatologic (acne, hirsutism), metabolic (insulin resistance, obesity, metabolic syndrome, type 2 diabetes, and dyslipidaemia) and psychological (depression, anxiety, poor self-esteem, body image concerns, and mental health disorders) (Wild et al., 1985; Coffey and Mason, 2003; Boomsma et al., 2006; Deek et al., 2010; Stepto et al., 2013; Teede et al., 2013; Joham et al., 2015). As the precise aetiology of PCOS remains partially unknown, a precise treatment approach has not been established and continues to be the subject of research and scientific inquiry. Currently, treatment of PCOS depends primarily on the desired goal. This may be to increase fertility, improve menstrual regularity, reduce hyperandrogenism and/or metabolic disorders and its consequences, including obesity. For this reason, the main therapeutical approaches are mainly related to physical exercise, hypocaloric diet, birth control pills, antiandrogens, and metformin (Graff et al., 2016; Spritzer et al., 2016; Tan et al., 2016; Xu et al., 2017; Jin and Xie, 2018; Barrea et al., 2019; Paoli et al., 2020; Woodward et al., 2020; Oguz and Yildiz, 2021; Szczuko et al., 2021; Armanini et al., 2022; Carmina et al., 2022; Gu et al., 2022; Rashid et al., 2022). In the last decade, botanicals, especially inositols and berberine, administered alone, in combination or as add-ons to pharmacological therapy, have proven to be effective in the management of women with PCOS (Wei et al., 2012; Fruzzetti et al., 2017; Zhao et al., 2021; Mishra et al., 2022). It is worth noting that the isoquinoline alkaloid berberine can interfere with the development of PCOS by alleviating insulin resistance, reducing the level of serum androgen and by regulating lipid metabolism and mild chronic inflammation (Li et al., 2018; Xi et al., 2019; Rondanelli et al., 2020; Zhang et al., 2021; Wang et al., 2021). Berberine is an “old” botanical discovered in the 1930s (Cromwell, 1933) and was effectively used, mainly in Asian countries, from the 1950s to the 1970s as an anti-dysentery treatment thanks to its action against enteric pathogens, on opioid μ e δ receptors and to its gut anti-secretory role (Chang, 1959; Homma et al., 1961; Mekawi, 1966; Lahiri and Dutta, 1967; Amin et al., 1969; Chauhan et al., 1969; Sharda, 1970; Zhang et al., 2012; Chen et al., 2015). In some Asian countries it was subsequently abandoned in favour of better-performing and perhaps less expensive anti-diarrhoeal drugs but it is still a commonly used first-line anti-diarrhoea drug in China. Later, it was re-discovered mainly as anti-cholesterol and anti-diabetic agent, thanks to peculiar mechanisms of action such as its effects on LDL and insulin receptors, and on PCSK9 (Proprotein convertase subtilisin/kexin type 9) and AMPK (AMP-activated protein kinase) (Kong et al., 2004; Lee et al., 2007; Yin et al., 2008; Zhu et al., 2019; Ataei et al., 2022). However, the good metabolic effects exerted by berberine are limited by its poor kinetic profile (Xu et al., 2021). To overcome berberine’s poor oral bioavailability, many formulative attempts have been made, including those aimed at increasing its solubility, those exploiting substances with P-gp inhibitor role or absorption enhancers, lipid-based formulations, formulations targeting lymphatic transport and physicochemical modifications increasing lipophilicity (Murakami et al., 2023a; Murakami et al., 2023b). Recently, attempts to improve the pharmacokinetics properties of berberine while maintaining tolerability, resulted in the development of a solid dispersion containing berberine, sunflower lecithin, pea protein, and grape seed oligomeric proanthocyanidin. The product is named Berberine Phytosome^®^ (BP). When administered to healthy volunteers, absorption is improved by up to ten-fold in comparison with pure berberine (Petrangolini et al., 2021). BP, in a small pilot study performed on 12 Caucasian, PCOS-diagnosed women, has demonstrated a significant decrease in Homeostasis Model Assessment (HOMA), C-Reactive Protein (CRP), TNF-α, triglycerides, testosterone, Body Mass Index (BMI), Visceral Adipose Tissue (VAT), fat mass, and acne severity, along with a significant increase in sex hormone-binding globulin (SHBG) (Rondanelli et al., 2021). Moreover, in human trials no side-effects, including the recognized gastrointestinal adverse effects of traditional berberine, have been reported, thus optimizing its tolerability even in long-term use. With these premises we decided to investigate the effect of BP on Asian (Pakistan) women. The study was completed in May 2023. This report is the detailed analysis of what was obtained.

## Materials and methods

This controlled, randomized, multi-centric and open-label clinical trial has been registered on https://clinicaltrials.gov/(identifier: NCT05480670) and approved by the Institutional Review Board (Ethics approval number: Ref. No. RC-2022/EA-01/178). The study aimed to evaluate the pharmacological and clinical role played by a food supplement containing berberine on women affected by PCOS. The women had all been enrolled at the gynaecological departments of different hospitals (Ayub Teaching Hospital, Bolan Medical Complex Hospital, King Edward Medical University, Khyber Teaching Hospital, Lady Reading Hospital). This study has been conducted in compliance with the guidelines of the Declaration of Helsinki and Good Clinical Practice. Informed written consent in their local language was obtained from each participant before enrolling in the study. All women were assured that declining to participate in the study or leaving the study at any point would not affect the quality of their treatment and that they would thereafter receive the best care available. The trial was performed between November 2022 and May 2023.

**FIGURE 1:**
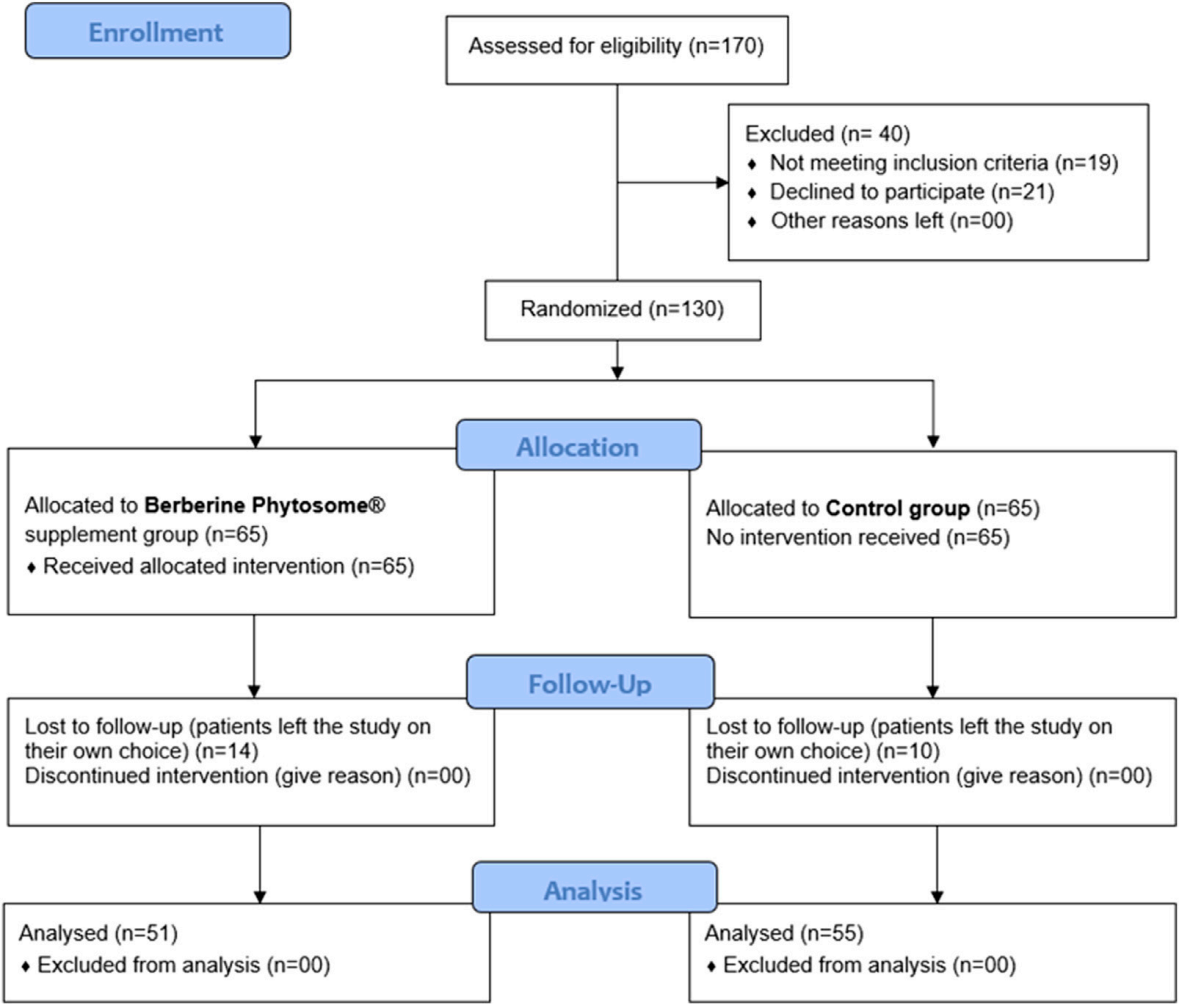
Flow Chart of the study.

Inclusion criteria were: women aged between 18 and 45 years, PCOS diagnosis as per the Rotterdam Criteria (Dokras et al., 2018), normal weight, overweight and obese with a BMI between 18.5 and 35. Exclusion criteria were: (i) women who are currently taking or who have recently stopped medications known to alter insulin physiology, oral contraceptives, ovulation induction drugs, anti-obesity or cholesterol lowering drugs, or any dietary supplement known to interfere with insulin and androgen metabolism (inositols, curcumin, lipoic acid, cinnamon, *etc.*); (ii) women undergoing *in vitro* fertilization treatment; (iii) women diagnosed with late-onset congenital adrenal hyperplasia, thyroid disorders, hyperprolactinemia, diabetes mellitus in the light of previous medical record; (iv) the presence of severe liver or renal disease; (v) pregnant, lactating or menopausal women; (vi) having anti-mullerian hormone (AMH) < 0.7. Drinking alcohol is legally banned in Pakistan therefore this aspect was not inserted in the exclusion criteria. Furthermore, all enrolled women denied smoking. Enrolled participants were randomly assigned by the treating physicians into two groups, the supplemented group (berberine group, indicated in tables and figures as B) and the control group (control group, indicated in tables and figures as C) in a 1:1 ratio. Randomization was carried out by a computer-generated random numbers code with odd numbers allocated to the participants in the berberine group and even numbers assigned to participants in the control group. According to the flow chart of the study (Figure 1), 170 women were assessed for eligibility and 130 were enrolled and randomized (65 per group). Fifty-one of the berberine group and 55 of the control group completed the study and were analysed.

The pre-enrolment analysis of dietary styles among the subjects participating in the study demonstrated the absence of significant differences. Moreover, on average the same number of subjects in the two groups declared non-celiac gluten sensitivity, lactose and/or FODMAPs (Fermentable Oligosaccharides, Disaccharides, Monosaccharides and Polyols) intolerance, and histamine and/or nickel intolerance (data not shown). All participants of both groups received the same standard of care advice. This standard of care advice corresponded to a modest reduction in dietary carbohydrates in the context of a weight-maintenance diet as described by Gower BA et coll (Gower et al., 2013). All participants were also advised to continue with their usual exercise, adding, if needed, cycling and walking. Those included in the berberine group were supplemented for 90 days with nutraceutical-grade berberine (see below for details) as an add-on therapy to their standard of care advice. No supplementation was suggested to the control group. As regards follow-up, each participant underwent four medical consultations. A baseline/enrolment in-person visit, a telephone consultation at day 30 and day 60 to check for supplementation adherence and side-effects, and an end-of-treatment (after 90 days) in-person visit. Participants’ study data were only recorded at baseline (before the treatment) and after completion of the 3-month treatment.

The berberine group were supplemented with Sophy^®^, a nutraceutical supplement kindly provided by Pharmextracta S.p.A., Pontenure (Italy) and notified to the Italian Health Authorities on 16 February 2022, document number 147496. Treatment corresponded to one tablet in the morning (after breakfast) and one in the evening (after dinner). The active ingredient in the nutraceutical product is Berberine Phytosome^®^ (BP, 550 mg/tablet, kindly provided by Indena S.p.A., Milan, Italy; Patent Application WO2019/150225). BP is a solid dispersion containing berberine extract from *Berberis aristata* formulated in sunflower lecithin, pea protein, and grape seed extract as previously described.^60^ BP is standardized to contain 28%–34% of berberine by HPLC (High Performance Liquid Chromatography), corresponding to 180 mg per each tablet in use in this study. Adherence to treatment was assessed by counting the number of supplements remaining at the end of the 90 days of treatment. All participants in the berberine group who ended the study returned less than 10% of the tablets provided.

### Outcomes

The primary outcome of the current study was an improvement in any single abnormality as identified in the Rotterdam Criteria: oligomenorrhea or amenorrhea; signs and/or symptoms of hyperandrogenism; polycystic ovaries visible on an ultrasound. Secondary outcomes were: (i) anthropometric measurements such as weight, BMI, waist circumference (W), hip circumference (H), and W/H ratio; (ii) sex hormone profile, such as free testosterone (FT), LH, follicle stimulating hormone (FSH) and AMH; (iii) glycaemic profile, such as fasting glucose (FG), fasting insulin (FI), haemoglobin A1C (HbA1C) and insulin resistance assessed by Homeostasis Model Assessment (HOMA) (Haffner et al., 1996); (iv) lipidic profiles, such total cholesterol (TC), triglycerides (TG), high density lipoprotein-cholesterol (HDL-C), low density lipoprotein-cholesterol (LDL-C); (v) non-invasive markers of liver injury and inflammation, such alanine aminotransferases (ALT), aspartate aminotransferases (AST), γ -Glutamyl Transferase (γ -GT), bilirubin, alkaline phosphatase (ALP), liver ultrasound for grading Non-Alcoholic Fatty Liver Disease (NAFLD), C-Reactive Protein (CRP); (vi) evaluation of the quality of life (QoL); (vii) safety (complete blood analysis) and side-effects (by questionnaire). Biochemical measurement methods are reported in Supplementary Material S1. QoL was evaluated at baseline and after 90 days of treatment by asking all enrolled women to complete a specific and validated questionnaire. The questionnaire had a total of 26 questions and determined PCOS effects on QoL across five different domains including emotions, body hair, body weight score (BWS), infertility, and menstrual problems. The score for each domain was calculated by summing different questions randomly distributed in the questionnaire. For emotions: the sum of scores of questions 2, 4, 6, 11, 14, 17, 18, 20. For body hair: the sum of scores of questions 1, 9, 15, 16, 26. For BWS: the sum of scores of questions 3, 10, 12, 22, 24. For infertility problems: the sum of scores of questions 5, 13, 23, 25. For menstrual problems: the sum of scores of questions 7, 8, 19, 21 (Cronin et al., 1998). The evaluation of the acne condition was performed by a dermatologist who calculated the severity of acne through the combined assessment of the types of acne lesions (comedones, papules, pustules, and nodules) and their anatomic location (forehead, cheeks, nose, and chin). Each type of acne lesion is given a value depending on severity: no lesions = 0, comedones = 1, papules = 2, pustules = 3, and nodules = 4. Each of the location was graded separately on 0–4 scale, with the most severe lesion within that location determining the local score. The severity was then graded according to the global score, which is the summation of all local scores. A score of 1–18 was considered mild/moderate; a score 19–32 was considered severe. Finally, for a further and more precise evaluation of hirsutism, we evaluated all participants with the modified Ferriman-Gallwey (mFG) score (Ilagan et al., 2019). All data was recorded in paper-based case report forms and transferred to an Excel file.

### Sample size calculation and statistical analysis

The sample size was determined through a power analysis aimed at estimating the minimum number of participants required to detect significant differences between groups with an acceptable degree of statistical power. To conduct the power analysis, we specified the following key parameters: 1) significance alpha-level [0.05]; 2) statistical 1-beta-power [0.80]; 3) minimum detectable size effect [Cohen’s d = 0.5]; and estimated sigma-standard deviation [σ = 10]. Using these parameters, we utilized a statistical software (G*Power) to calculate the minimum number of participants required to achieve a statistical power of 80%. The result of the power analysis indicated that a sample size of at least 106 subjects was needed to detect significant differences with the specified minimum detectable effect and the established levels of significance and power. We have then adopted a conservative approach to ensure that the study had sufficient statistical power to detect the anticipated differences also considering the potential loss due to withdrawals. Our sample size calculation approach agreed with what was done in a previous study carried out to highlight the clinical role exerted by berberine on women with PCOS (Orio et al., 2013).

As regards to statistics, comparisons of quantitative baseline variables were performed using Confidence Intervals for proportion, with alpha = 0.05. The comparisons of qualitative variables, including recruitment centre, type of family unit, residence, occupation, ongoing medical therapies, reason for failure to conceive, previous miscarriages and possible depressive state caused by the condition of polycystic ovary, the analysis of contingency tables using chi-square tests was used, with alpha = 0.05. The anthropometric variables, such as weight (Kg), height (cm), BMI (Kg/m^2^), waist circumference (cm), hip circumference (cm) and waist to hip ratio (WHR), were analysed at 90 days *versus* the baseline, using Split-Plot ANOVA and Tukey’s multiple comparison test for non-confounded means. The same ANOVA was used for the analysis of sex hormones, glycaemic, lipidic and liver profiles. The QoL scores for PCOS and the complete blood analysis for safety were also analysed using Split-Plot ANOVA. To compare groups and the time periods of variables such as menstrual cycle status, pelvic ultrasound results, presence of acne and presence of hirsutism, the JMP 14 Categorical software from SAS Institute was used. The anonymous raw data and the performed statistical analysis are available on reasonable request.

## Results

### Quantitative, qualitative and anthropometric features

**FIGURE 2:**
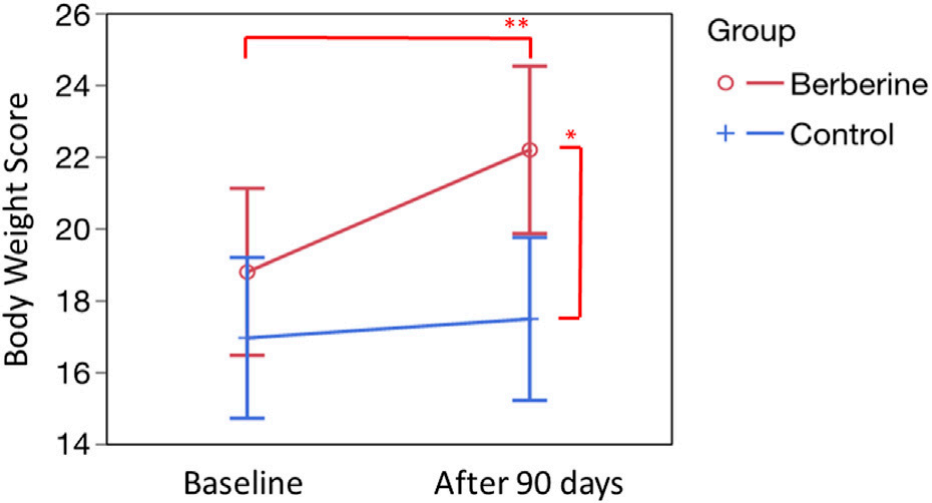
Evaluation of the Body Weight score (BWS). BWS was calculated according to the specific questions proposed in the questionnaire by Cronin et al. (Cronin et al., 1998). Questions are four: 1) During the past 2 weeks, how much of the time you had trouble dealing with your weight? 2) During the past 2 weeks, how much of the time you felt frustration in trying to lose weight? 3) How much of the time during the last 2 weeks did you feel like you are not sexy because of being overweight? 4) How much of the time during the last 2 weeks did you have difficulties staying at your ideal weight? Basal scores, expressed as mean ± standard error, were 17.78 ± 1.74 (Berberine) and 16.94 ± 1.13 (Control). After 90 days of treatment, were 22.18 ± 1,18 (Berberine; *p* = 0.0260 *versus* control at 90 days, see single asterisk; and *p* = 0.0016 *versus* basal value, see double asterisk) and 17.47 ± 1.48 (Control; not significant *versus* basal value). Statistics was obtained by Tukey’s test.

As reported in the flow chart (Figure 1), out of 170 women assessed for eligibility, 19 women were excluded because they did not meet the inclusion criteria and 21 women because they declined to participate. Of 130 randomized participants, 14 in the berberine group and 10 in the control group left (of their own choice) during follow-up; 106 women completed the study. At enrolment, most quantitative and qualitative features, were not significantly different (Table 1; Table 2) between the two groups (Berberine and Control). At baseline, some anthropometric measurements, such as body weight, BMI, waist, and hip measurements, were slightly higher in the control group despite randomization, but were found not to be significantly different (Table 3). Regarding the anthropometric measurements, after 90 days of supplementation, with the exception of the WHR, where a significant result was achieved only in the berberine group, the parameters did not appear to follow a treatment-specific trend. In fact, weight, BMI, waist and hip circumferences demonstrated completely superimposable trends due to a possible greater impact of the common dietary change proposed to the participants of both groups (Table 3). Nevertheless, the Body Weight Score (BWS) demonstrated an exclusively significant increase for the berberine group (Figure 2). Indeed, although both the berberine group and the control group lost weight during the 90 days of the study (Table 3), the weight loss observed in the berberine group was greater than that observed in the control group (5.19% *versus* 3.05%).

**TABLE 1:** Study participant’s quantitative baseline data, expressed as mean ± standard deviation.

Parameter	Berberine group (n = 51)	Control group (n = 55)	p
Age (years)	24.2 ± 5.0	24.8 ± 6.2	n. s
SBP (mmHg)	111.8 ± 13.2	114.1 ± 11.7	n. s
DBP (mmHg)	71.5 ± 13.0	75.7 ± 8.2	n. s
Pulse rate (bpm)	81.3 ± 6.5	83.0 ± 6.3	n. s
Non-conceiving (months)	48.9 ± 39.5	44.8 ± 30.8	n. s

SBP, systolic blood pressure; DBP, diastolic blood pressure; bpm, beats per minute; n. s., not significant.

**TABLE 2:** Baseline qualitative data of participants in the two groups, berberine (B; n = 51) and control (C; n = 55).

Group/Variable	Category	Numbers	%
B/Recruitment Center	ATH	16	31.373
	BMCH	10	19.608
	KEMU	15	29.412
	KTH	0	0.000
	LRH	10	19.608
C/Recruitment Center	ATH	16	29.091
	BMCH	4	7.273
	KEMU	16	29.091
	KTH	9	16.364
	LRH	10	18.182
B/Marital Status	Divorced	2	3.922
	Married	26	50.980
	Single	23	45.098
C/Marital Status	Divorced	1	1.818
	Married	31	56.364
	Single	23	41.818
B/Number of children	0	19	37.255
	1	3	5.882
	2	3	5.882
	3	2	3.922
	Not applicable	24	47.059
C/Number of children	0	18	32.727
	1	6	10.909
	2	2	3.636
	3	1	1.818
	4	2	3.636
	5	1	1.818
	7	1	1.818
	Not applicable	24	43.636
B/Residence	Rural	17	33.333
	Urban	34	66.667
C/Residence	Rural	24	43.636
	Urban	31	56.364
B/Occupation	Community worker	1	1.961
	Medical doctor	2	3.922
	Housewife	19	37.255
	Maid	2	3.922
	Nurse	1	1.961
	Polio worker	1	1.961
	Software engineer	1	1.961
	Speech therapist	2	3.922
	Staff nurse	1	1.961
	Student	18	35.294
	Teacher	1	1.961
	Unemployed	2	3.922
C/Occupation	Dentist	2	3.636
	Health worker	2	3.636
	Housewife	29	52.727
	Manager	1	1.818
	Nurse	1	1.818
	Pharmacist	1	1.818
	Speech therapist	1	1.818
	Student	18	32.727
B/Medication	None	50	98.039
	Trimebutine	1	1.961
C/Medication	None	54	98.182
	Solifenacin	1	1.818
B/Reason for not conceiving (self-declared)	Not available	25	49.020
	Not known	2	3.922
	Not sure	2	3.922
	Menses not regular	7	13.725
	PCOS	13	25.490
	Hormonal imbalance	1	1.961
	Overweight	1	1.961
C/Reason for not conceiving (self-declared)	Not available	27	49.091
	Not known	1	1.818
	Not sure	13	23.636
	Menses not regular	3	5.454
	PCOS	11	20.000
B/Previous Miscarriage	No	44	86.274
	Yes	7	13.726
C/Previous Miscarriage	No	49	89.091
	Yes	6	10.909
B/Depressive mood due to PCOS	No	8	15.686
	Yes	43	84.314
C/Depressive mood due to PCOS	No	17	30.909
	Yes	38	69.091

The analysis of the contingency tables using the chi-square test demonstrated the absence of difference between the two groups of enrolled subjects for the variables considered. ATH, Ayub Teaching Hospital; BMCH, Bolan Medical Complex Hospital; KEMU, King Edward Medical University; KTH, Khyber Teaching Hospital; LRH, Lady Reading Hospital.

**TABLE 3:** Anthropometric descriptive statistics, expressed as mean ± standard error, of the participants in the two groups, berberine (B; n = 51) and control (C; n = 55), at enrolment and after 90 days of treatment.

Group/Parameter	Baseline	After 90 days	p*
B/Body weight (kg)	69.06 ± 2.14	65.48 ± 2.13	<0.001
C/Body weight (kg)	76.32 ± 2.06	73.99 ± 2.07	<0.001
B/BMI (kg/m^2^)	27.29 ± 0.86	25.83 ± 0.85	<0.001
C/BMI (kg/m^2^)	30.14 ± 0.83	29.23 ± 0.85	<0.001
B/Waist circumference (cm)	89.92 ± 1.85	90.29 ± 1.85	n. s
C/Waist circumference (cm)	94.75 ± 1.79	94.64 ± 1.79	n. s
B/Hip circumference (cm)	100.84 ± 1.87	98.89 ± 1.87	<0.001
C/Hip circumference (cm)	104.34 ± 1.80	102.55 ± 1.80	<0.001
B/WHR	0.899 ± 0.02	0.919 ± 0.02	n. s
C/WHR	0.915 ± 0.02	0.927 ± 0.02	n. s

Statistics calculated as a comparison between “baseline” and between “after 90 days” values demonstrated not significant differences. *statistics calculated as “after 90 days” *versus* “baseline”. BMI, body mass index; WHR, waist to hip ratio; n. s., not significant.

### Biochemical analysis

As far as the glucose and lipid asset is concerned (Table 4), both groups showed a decreasing trend. However, in most cases this appeared not to be significant, as in the case of the HOMA (Homeostatic Model Assessment index) (data not shown), fasting glycemia, insulin, glycated haemoglobin, HDL, and LDL. Nonetheless, the trend was significant in relation to total cholesterolemia and triglyceridemia. In the latter case, however, the significance concerned only the control group. It was a slightly different story for the framework relating to the liver enzymes (AST: Aspartate Transaminase; ALT: Alanine Transaminase; ALP: Alkaline Phosphatase; γ-GT: γ -Glutamyl Transferase), for bilirubin and for CRP (C-Reactive Protein). In fact, for these parameters the downward trend, albeit again slight and not significant, was in any case only observable in the berberine group. By contrast, in some cases, a moderate increase was observed in the control group (Table 5). As far as the hormonal balance is concerned, although significant differences were not observed, the berberine group showed a better trend in values than in the control group, with a slight increase in AMH, a decreasing trend for testosterone and a slight decrease in FSH. LH was substantially unchanged (Table 6). About 50% of the women enrolled in the study were also given an abdominal ultrasound to estimate the fatty infiltration of the liver. As shown in Table 7, an improvement trend, while not significant, was observed in the liver of both groups with a very slightly more favourable trend in the berberine group.

**TABLE 4:** Metabolic descriptive statistics, expressed as mean ± standard error, of participants in the two groups, berberine (B; n = 51) and control (C; n = 55), at enrolment and after 90 days of treatment.

Group/Parameter	Baseline	After 90 days	p*
B/Fasting glucose (mg/dL)	86.24 ± 2.42	85.62 ± 2.42	n. s
C/Fasting glucose (mg/dL)	90.03 ± 2.32	88.91 ± 2.32	n. s
B/Fasting insulin (µUI/mL)	36.99 ± 5.89	36.77 ± 5.91	n. s
C/Fasting insulin (µUI/mL)	39.78 ± 5.77	36.14 ± 5.67	n. s
B/Hb A1C (%)	5.25 ± 0.15	5.12 ± 015	n. s
C/Hb A1C (%)	5.45 ± 0.15	5.22 ± 0.15	n. s
B/TC (mg/dL)	166.44 ± 6.32	155.41 ± 6.32	0.0192
C/TC (mg/dL)	180.68 ± 5.66	166.14 ± 5.66	0.0019
B/TG (mg/dL)	136.73 ± 11.03	118.23 ± 11.11	n. s
C/TG (mg/dL)	145.91 ± 10.62	119.15 ± 10.62	0.0433
B/HDL (mg/dL)	44.41 ± 1.82	43.97 ± 1.84	n. s
C/HDL (mg/dL)	41.69 ± 1.75	43.95 ± 1.75	n. s
B/LDL (mg/dL)	111.14 ± 4.37	104.80 ± 4.39	n. s
C/LDL (mg/dL)	113.99 ± 4.31	105.76 ± 4.21	n. s

Statistics calculated as a comparison between “baseline” and between “after 90 days” values demonstrated not significant differences. *statistics calculated as “after 90 days” *versus* “baseline”. Hb A1C, glycated haemoglobin; TC, total cholesterol; TG, triglycerides; HDL, high density lipoprotein; LDL, low density lipoprotein; n. s., not significant.

**TABLE 5:** Liver Enzymes and CRP descriptive statistics, expressed as mean ± standard error, of participants in the two groups, berberine (B; n = 51) and control (C; n = 55), at enrolment and after 90 days of treatment.

Group/Parameter	Baseline	After 90 days	p*
B/AST (U/L)	25.22 ± 2.64	24.85 ± 2.64	n. s
C/AST (U/L)	24.91 ± 2.52	28.04 ± 2.52	n. s
B/ALT (U/L)	32.11 ± 3.55	30.09 ± 3.55	n. s
C/ALT (U/L)	36.78 ± 3.32	39.82 ± 3.32	n. s
B/Bilirubin (mg/dL)	0.60 ± 0.03	0.52 ± 0.03	n. s
C/Bilirubin (mg/dL)	0.56 ± 0.03	0.56 ± 0.03	n. s
B/ALP (U/L)	129.82 ± 11.45	118.85 ± 11.45	n. s
C/ALP (U/L)	124.51 ± 11.03	137.67 ± 11.03	n. s
B/γ-GT (U/L)	24.95 ± 2.37	20.31 ± 2.37	n. s
C/γ-GT (U/L)	20.58 ± 2.29	24.37 ± 2.29	n. s
B/CRP (U/L)	2.29 ± 0.47	2.01 ± 0.47	n. s
C/CRP (U/L)	2.30 ± 0.45	2.69 ± 0.45	n. s

Statistics calculated as a comparison between “baseline” and between “after 90 days” values demonstrated not significant differences. *statistics calculated as “after 90 days” *versus* “baseline. AST, alanine aminotransferases; AST, aspartate aminotransferases; ALP, alkaline phosphatase; γ-GT, γ -Glutamyl Transferase; CRP, C Reactive Protein; n. s., not significant.

**TABLE 6:** Hormonal descriptive statistics, expressed as mean ± standard error, of participants in the two groups, berberine (B; n = 51) and control (C; n = 55), at enrolment and after 90 days of treatment.

Group/Parameter	Baseline	After 90 days	p*
B/AMH (ng/mL)	5.00 ± 0.39	5.16 ± 0.39	n. s
C/AMH (ng/mL)	4.80 ± 0.37	4.07 ± 0.37	n. s
B/Free-T (ng/dL)	7.25 ± 2.09	5.55 ± 2.09	n. s
C/Free-T (ng/dL)	5.99 ± 2.01	5.76 ± 2.01	n. s
B/FSH (UI/L)	6.78 ± 1.36	6.09 ± 1.35	n. s
C/FSH (UI/L)	7.13 ± 1.31	9.93 ± 1.31	n. s
B/LH (UI/L)	9.88 ± 1.33	10.87 ± 1.33	n. s
C/LH (UI/L)	15.31 ± 1.28	14.37 ± 1.28	n. s

Statistics calculated as a comparison between “baseline” and between “after 90 days” values demonstrated not significant differences. *statistics calculated as “after 90 days” *versus* “baseline. AMH, Anti-Mullerian Hormone; T, testosterone; FSH, Follicle-Stimulating Hormone; LH, luteinizing hormone; n. s., not significant.

**TABLE 7:** Ultrasound imaging to estimate fatty infiltration of the liver in participants in the two groups, berberine (B) and control (C), at enrolment and after 90 days of treatment.

Liver ultrasound	B–baseline	C–baseline	B–90 days	C–90 days
**Number of subjects,** *n*	26	35	25	32
**Normal,** *n (%)*	21 (80.8%)	21 (60.0%)	23 (92.0%)	25 (78.1%)
**NAFLD,** *n (%)*	5 (19.2%)	14 (40.0%)	2 (8.0%)	2 (8.0%)
**p** between “baseline” values: 0.0832 between “90 days” values: 0.1413				

NAFLD, Non-Alcoholic Fatty Liver Disease.

### Menstrual cycle, ovaries and infertility

Regarding the menstrual cycle status (Table 8), berberine supplementation restored regularity in 35 out of 45 women (70%; *p* < 0.0001). This compares to just 9 out 50 women in the control group (16.4%; not significant). It was possible to statistically analyse this result by reducing the different diagnoses of the participants (provided descriptively by the physicians responsible for the study) to simpler parameters such as “Normal”, “Abnormal” and “Improved” (Full details on the reasoning for this simplification of terminology and the statistical analysis adopted is available in Supplementary Materials S2, S3) A similar simplification was adopted for the ovarian ultrasound analysis results. Indeed, in this case, it was necessary to simplify descriptive diagnoses using the standard terms ‘normal’, ‘abnormal’, ‘improved’ for the purposes of statistical analysis (see Supplementary Materials S4, S5). An abnormal ovarian condition, which was present at enrolment in 90% of the women in the berberine group, significantly decreased to a value around 30% over the 90 days of treatment. In contrast, in the control group, the same ovarian condition, similarly present in about 90% of the women at enrolment, was improved or tending towards normality in approximately 20% of the women (Table 9). Regarding infertility and menstrual cycle problems, both analysed by the questionnaire, the scores from the berberine group demonstrated a tendential but not significant improvement of the values after treatment *versus* baseline values while the control group recorded a mild, but not significant, worsening in the scores for infertility problems *versus* baseline scores after supplementation and a not significant improvement in menstrual cycle problems, which is inferior when compared to that observed in the berberine group (data not shown).

**TABLE 8:** Menstrual cycle status of participants in the two groups, berberine (B) and control (C), at enrolment and after 90 days of treatment.

Menstrual status	B–baseline	C–baseline	B–90 days	C–90 days
**Number of subjects** *, n*	51	55	50*	55
**Normal,** *n (%)*	6 (11.8%)	5 (9.1%)	6 (12.0%)	4 (7.3%)
**Abnormal** *, n (%)*	45 (88.2%)	50 (90.9%)	9 (18.0%)	41 (74.5%)
**Improved,** *n (%)*	0 (0%)	0 (0%)	35 (70.0%)	9 (16.4%)
**p** between “baseline” values: 0.6520 between “90 days” values: <0.0001				

Normal, 4–7 days of bleeding every 24–36 days. Abnormal, from 2 to 3 days of bleeding every 2 months to 4–8 days of bleeding every 6 months. Improved, normalized. The detailed menstrual cycle data of all participants are available in Supplementary Material S2. For detailed statistics (Likelihood Ratio, Pearson, and Fisher) see Supplementary Material S3. *For 1 participant of the Berberine group the menstrual cycle data after 90 days of treatment was not available.

**TABLE 9:** Pelvic ultrasound findings of participants in the two groups, berberine (B) and control (C), at enrolment and after 90 days of treatment.

Pelvic ultrasound	B–baseline	C–baseline	B–90 days	C–90 days
**Number of subjects,** *n*	51	55	51	55
**Normal,** *n(%)*	5 (9.8%)	2 (3.6%)	4 (7.8%)	1 (1.8%)
**Abnormal,** *n(%)*	46 (90.2%)	53 (96.4%)	14 (27.5%)	40 (72.7%)
**Improved,** *n(%)*	0 (0%)	0 (0%)	33 (64.7%)	14 (25.5%)
**p** between “baseline” values: 0.1959 between “90 days” values: <0.0001				

Normal, normal ovary. Abnormal, Mono or bilateral PCOS, with or without stroma thickness. Improved, normalized or from bilateral to mono-lateral PCOS., The detailed pelvic ultrasound data of all participants are available in Supplementary Material S4. For detailed statistics (Likelihood Ratio, Pearson, and Fisher) see Supplementary Material S5.

### Acne and hirsutism evaluation

**FIGURE 3:**
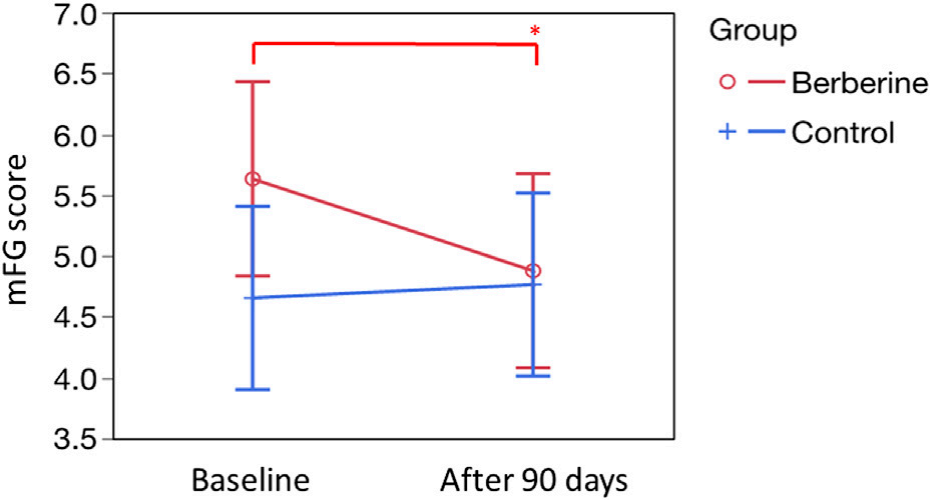
Evaluation of hirsutism, according to the modified Ferriman-Gallwey (mFG) score. Basal scores, expressed as mean ± standard error, were 5.63 ± 0.40 (Berberine) and 4.65 ± 0.38 (Control). After 90 days of treatment, were 4.87 ± 0.40 (Berberine; *p* = 0.0152 *versus* basal value, see single asterisk) and 4.65 ± 0.38 (Control; not significant *versus* basal value). Statistics was obtained by Tukey’s test.

Regarding the more “aesthetic” aspect of PCOS, but still an important source of distress for women with PCOS, the presence of acne and the manifestations of hirsutism were evaluated. As shown in Table 10, berberine supplementation significantly reduced the presence of acne in approximately 50% of cases. No such significant results were recorded in the control group (Table 10) (Also in this case, for an accurate examination of the appearance of acne in each individual woman and a precise evaluation of the calculations performed for statistical purposes, please refer to Supplementary Materials S6, S7) To evaluate the presence and the trend of hirsutism throughout the study, we adopted the modified Ferriman-Gallwey (mFG) score. Regarding basal scores, the berberine group showed a higher value (5.63 ± 0.40 *versus* 4.65 ± 0.38 in the control group; not significant). Noteworthy, after 90 days of supplementation, the value in the berberine group dropped significantly to 4.87 ± 0.40 (*p* = 0.0152), while the value in the control group was unmodified (Figure 3). Similarly, the women’s body hair conditions, subjectively evaluated by the questionnaire, showed an improved trend only in the berberine group (data not shown).

**TABLE 10:** Presence of acne in the participants in the two groups, berberine (B) and control (C), at enrolment and after 90 days of treatment.

Presence of acne	B–baseline (n = 51)	C–baseline	B–90 days	C–90 days
**Number of subjects,** *n*	51	55	51	55
**Yes,** *n (%)*	29 (56.9%)	28 (50.9%)	13 (25.5%)	19 (34.5%)
**Mild,** *n (%)*	0 (0.0%)	0 (0.0%)	0 (0.0%)	1 (1.80%)
**No,** *n (%)*	22 (43.1%)	27 (49.1%)	26 (51.0%)	30 (54.5%)
**Improved,** *n (%)*	0 (0.0%)	0 (0.0%)	12 (23.5%)	5 (9.1%)
**p** between “baseline” values: 0.5389 between “90 days” values: *p* < 0.05				

The detailed acne data of all participants are available in Supplementary Material S6. For detailed statistics (Likelihood Ratio, Pearson, and Fisher) see Supplementary Material S7.

### Wellbeing evaluation

**FIGURE 4:**
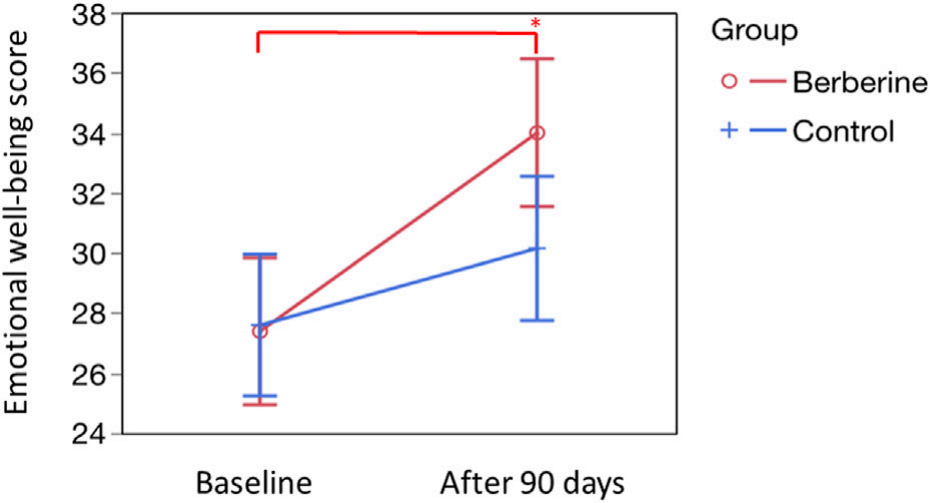
Evaluation of the emotional wellbeing score. The emotional wellbeing score was calculated according to the specific questions proposed in the questionnaire by Cronin et al. (Cronin et al., 1998). Question are eight: 1) During the past 2 weeks, how much of the time you felt depressed as a result of having PCOS? 2) During the past 2 weeks, how much of the time you felt easily tired? 3) During the past 2 weeks, how much of the time you felt moody as a result of having PCOS? 4) During the past 2 weeks, how much of the time have you had low self-esteem as a result of having PCOS? 5) During the past 2 weeks, how much of the time have you felt frightened of getting cancer? 6) During the past 2 weeks, how much of the time have you been worried about having PCOS? 7) During the past 2 weeks, how much of the time have you been self-conscious as a result of having PCOS? 8) In relation of your last menstruation, how much the following issues were a problem for you: late menstrual period? Basal scores, expressed as mean ± standard error, were 27.39 ± 1.24 (Berberine) and 27.60 ± 1.19 (Control). After 90 days of treatment, were 34.00 ± 1.24 (Berberine; *p* < 0.0001 *versus* basal value, see single asterisk) and 30.15 ± 1.21 (Control; not significant *versus* basal value). Statistics was obtained by Tukey’s test.

The emotional wellbeing score, subjectively analysed by the questionnaire, also showed a significant improvement only after berberine supplementation. In fact, as shown in Figure 4, basal scores, were 27.39 ± 1.24 for the berberine group and 27.60 ± 1.19 for the control group. After 90 days of supplementation, the berberine group value rose to 34.00 ± 1.24 (*p* < 0.0001) with no significant increase observed in the control group.

### Safety

**FIGURE 5:**
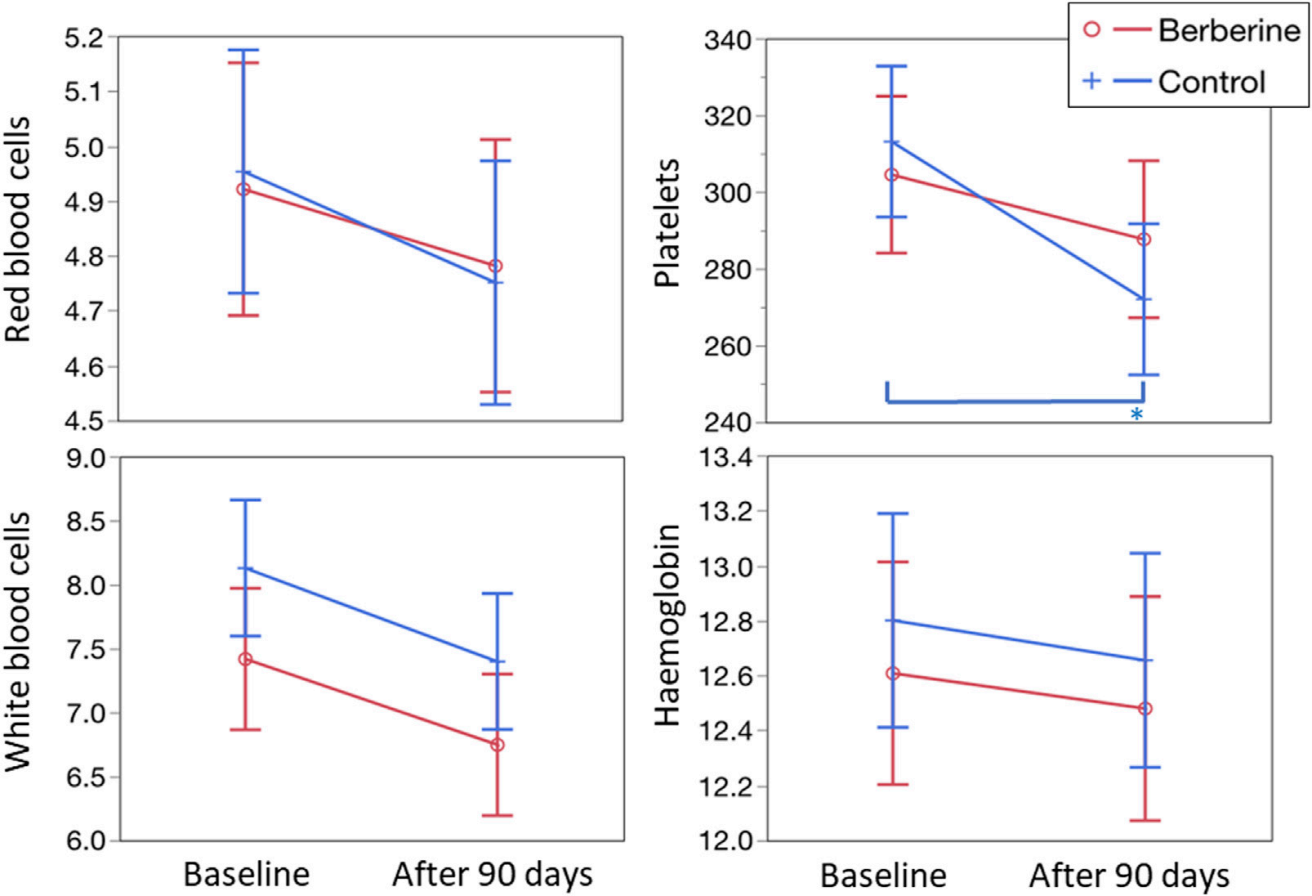
Evaluation of the main blood parameters. Values for red blood cells and platelets are expressed as 10^9^/L; values for white blood cells are expressed as 10^12^/L; values for haemoglobin are expressed as g/L. For all four parameters shown in the figure and for the haematocrit value (data not shown), no statistically significant differences, both between groups and between times, were observed. For platelets, the control group showed a significant decrease (*p* < 0.0005, see asterisk) at 3 months *versus* baseline.

Regarding safety, no significant modifications were observed in red and white blood cells, platelets, haemoglobin (Figure 5), haematocrit and mean cell volume (data not shown). Adverse events, registered in about 6% of the enrolled women during the study, were overlapping in the two groups both for type (constipation, flatulence, bloating, gastralgia, nausea, and headache), incidence (three women per group) and severity (mild and transient, lasting no more than 2 days each; data not shown).

## Discussion

Berberine is an isoquinoline alkaloid compound commonly extracted from *Coptis chinensis* and/or *B. aristata* (Cicero, 2016). Past research had mainly showed the anti-microbial and anti-diarrhoea actions of berberine (Khan et al., 2023). Modern research has proven that it has multiple pharmacological and clinical uses, including an effective role in improving glycaemic, lipidic and liver profiles of patients affected by metabolic diseases (Ye et al., 2021), especially when its low rate of absorption is improved by formulative approaches (Di Pierro et al., 2012; Di Pierro et al., 2013; Orio et al., 2013). Berberine oral bioavailability in animal and in human is indeed poor (Gasmi et al., 2023). Nonetheless, it is possible that, despite its poor pharmacokinetics, berberine could exert a certain anti-dysmetabolic role also by modulating the gut microbiome (Zhang et al., 2020).

A recent pilot study (Rondanelli et al., 2021), performed on twelve Caucasian women affected by PCOS, demonstrated that the administration of the same nutraceutical form of berberine (BP) we have used in our study. BP, which is formulated with phospholipids, pea proteins and procyanidolic oligomers from grape seeds in such a way as to produce an ameliorated plasma absorption and protect from eventual gastrointestinal discomfort in comparison to the administration of pure berberine (Petrangolini et al., 2021), can produce clear positive effects. This was evident from the analysis of the parameters commonly used as references in the diagnosis of PCOS, such as insulin resistance, lipid profile, hyperandrogenism (both biochemical and clinical) and inflammatory balance. However, the study in question had important limitations such as the small number of women observed and the absence of a control group. Since better dietary control can improve these parameters, at least in part, the absence of a control group did not provide an understanding of the extent to which dietary recommendations provided by health professionals influenced the results. However, in addition to the small number of participants and the absence of a control group, the pilot study enrolled women diagnosed with PCOS but with normal menstrual cycles. This further aspect limited the possibility of generalizing the conclusions drawn or extending them to a wider population including women with irregular cycles.

Our study therefore tried to overcome these main problems, enrolling a greater number of women with PCOS, designing a randomized and controlled study protocol, and evaluating women with important fertility problems due to irregular or absent, menstrual cycles. Of course, considering that our study was performed on an ethnically limited population, Pakistani, with differences in genetic polymorphisms and diet, our results may only have an indicative value for other populations such as the European ones.

The results of our study have shown that the improvement in anthropometric data (Table 3) is, for the most part, attributable to the dietary and habit suggestions provided to all the women enrolled in the study. In fact, the results obtained in the berberine and control groups, seem to show a parallel and significant decreasing trend. However, body weight (Table 3), despite a significant reduction in both groups (*p* < 0.001), showed a more evident percentage decrease in the group supplemented with berberine (5.2% *versus* 3.0%), and the subjective perception of weight loss (BWS, Figure 2) was significantly different (*p* = 0.0260 *versus* control at 90 days, and *p* = 0.0016 *versus* berberine basal value) only in the berberine group. This therefore supports a more favourable action played by the berberine supplementation at least on weight.

As far as the glycaemic and lipid profiles are concerned (Table 4), the effects observed in the two groups proved to be very similar with completely superimposable improvement trends except for the triglyceride parameter, where the decrease was more evident in the control group. In meta-analysis papers, berberine is reported to significantly improve both the glucose and the lipid profiles (Li et al., 2023). It is possible that the averaged low baseline values recorded at enrolment in women of both groups (indeed the vast majority of them were normoglycemic and normolipidemic) did not allow for observation of the expected hypoglycaemic and lipid-lowering effects.

Similarly, as regards the profile of liver enzymes and C-Reactive Protein (Table 5), the results did not prove to be significantly different between the two groups. Even in this case, however, the starting values could be considered “normal”. That said, once again the results still show a tendency towards lower values attributable more to supplementation with berberine.

The hormonal profile (Table 6) also showed non-significant variations in the two groups. However, it should be emphasized that the reduction in FT reached 23.5% in the berberine group and only 3.8% in the control one. This difference, although not significant, demonstrates the potentially antiandrogenic role observed in many other studies (Zhang et al., 2021). Serum testosterone levels are generally considered representative of ovarian androgen generation, while dehydroepiandrosterone (DHEA) and its sulphated ester (DHEAS) production is primarily reflective of adrenal androgen production. Debate still persists over the predominant source of androstenedione production, with likely relatively equivalent contributions from the adrenals and ovaries (Cussen et al., 2022). Some authors have also described the androstenedione lowering properties of berberine in PCOS (Wang et al., 2010; Mirzaee et al., 2021; Rondanelli et al., 2021). Perhaps an evaluation of its plasma profile would have allowed us to better understand the clinical antiandrogenic effects (menstrual and ovary normalization, anti-acne, and anti-hirsutism action), observed in our study.

Ultrasound is suggested as first-line screening tool for defining NAFLD (Papatheodoridi and Cholongitas, 2018). NAFLD is often observed in PCOS, especially in overweight or obese women (Anagnostis et al., 2018). The ultrasound imaging to estimate fatty infiltration of the liver in women from both groups, although performed in only about half of the participants (Table 7), showed a mild improvement in both groups with no appreciable differences, which is likely due to the small number of women with visible fatty liver infiltration at baseline (that is, 5 in the berberine group and 14 in the control one).

Oligo- and amenorrhea, are considered important clinical manifestations of PCOS and menstrual cycle control is necessary to prevent endometrial hyperplasia and infertility (Reiser et al., 2022). In our study, menstrual disorders were present in about 90% of the entire enrolled sample (Table 8). Berberine supplementation caused normalization in 70% of the supplemented women; effects attributable to the dietary approach alone were not more than 16% (value observed in the control group). While indirect, this is also a possible demonstration of the effect of berberine on the resumption of a certain ovarian physiology otherwise affected by hyperandrogenism (Rosenfield and Ehrmann, 2016).

The ultrasound survey of the ovaries allowed clear insight into abnormal conditions such as the presence of multiple cysts or of altered dimensions with mono- or bilateral characteristics, as well as structural anomalies in the stromal thickness. Its use constitutes a powerful tool to accurately diagnose PCOS and to associate it with metabolic and endocrine processes such as hyperandrogenism and insulin resistance (Giménez-Peralta et al., 2022). In our study, at baseline, more than 90% of the enrolled women showed ultrasound imaging of PCOS. Berberine supplementation significantly reduced these abnormal features from about 90% to 27% while, in the control group, the reduction was from 96% to 73% of the sample (Table 9).

As previously stated, acne and hirsutism can be considered as possible clinical evidence of hyperandrogenism. Berberine supplementation seems to have a particularly evident pharmacological effect capable of significantly reducing both acne and hirsutism in women with PCOS, as shown in Table 10 and in Figure 3. These aspects, along with the effects on menstrual cycle irregularity and ovarian abnormalities, are most likely responsible for the positive impact of berberine supplementation on the enhanced emotional state and feelings of wellbeing perceived by the women in the berberine group (Figure 4).

Overall, our results show the extent to which berberine, formulated in such a way as to safely reduce its poor oral absorption, can enhance the positive effects of following a nutritionally controlled nutritional diet. Our study demonstrates that, even in a relatively short period of time, it is possible to determine significant changes that effectively counteract the menstrual cycle irregularities, abnormalities in the ovarian structure and the phenotypic (cutaneous) manifestations of hyperandrogenism, thus determining a quality of life that is perceived as better. In our study, the glycaemic, lipid, hepatic and hormonal profiles were perhaps not sufficiently altered for an adjuvant treatment to an improved nutritional diet to have a significant impact on improving these parameters when compared to the control group alone. Regardless of why this occurred, berberine did not lead to clearly significant advantages when compared to diet alone but, at most, it revealed an apparent tendency to further improvement. Of course, we have wondered if a better berberine plasma profile could have negatively influenced the metabolic effects exerted by supplementation. Among the various mechanisms of action of berberine, there is that mediated by the incretin known as glucagon-like peptide 1 (GLP-1). It is well-known that berberine is a very bitter substance capable of activating the intestinal bitterness receptors. These in turn induce the release of GLP-1, among whose cascading effects there is also that exerted on satiety (Yu et al., 2015). Obviously, it is possible that a better oral absorption of berberine could result in not only a lower “microbiota-mediated” metabolic effect but also a lower “contact” with the bitter receptors. Obviously, our study had in no way envisaged the possibility of addressing such mechanistic aspects and therefore these remain entirely speculative considerations. At the same time, they cannot be excluded. From a clinical point of view, perhaps the simplest way to validate this type of hypothesis would be to supplement the alkaloid berberine by formulating it both in ways that make it more absorbed, for example, as in the phytosome form (BP) that we used in our study, and in pure form. Once the obvious galenical and dosage problems–which should be addressed–have been resolved, this could be the next investigative step to be performed.

In contrast to other authors who have reported gastrointestinal side-effects linked to berberine use in PCOS (Lause et al., 2017), no peculiar adverse events were observed in the berberine group in this study. The difference could be due to the formulation used, as previously observed (Rondanelli et al., 2021). Indeed, the bioavailable form of berberine (see Supplementary Material S8) allowed us to use a lower dose of the alkaloid per unit than that use in similar trials (Ma et al., 2011; An et al., 2014; Lause et al., 2017). Moreover, the innovative formulation was rationally developed with the aim to protect the intestinal mucosa against the known gastrointestinal adverse effects of traditional berberine, thus optimizing its tolerability even in long-term use (Petrangolini et al., 2021). The lack of side-effects is very important considering that this supplementation will be used for chronic conditions and that bowel discomfort is the main issue hampering the long-term use of berberine.

This study clearly has some limitations that cannot be denied. Certainly, the enrolled sample size, although not small, is not yet large enough to outline absolute certainties. Also, despite being a randomized and controlled trial, this was not performed under blind conditions or with a true placebo treatment. Finally, ours is certainly a study performed on an ethnically limited population, Pakistani, and therefore our conclusions should first be confirmed in other populations, such as the European one, before they can be extended worldwide. Neither does our study allow us to definitely outline the metabolic role of berberine when supplemented in phytosome. Indeed, the enrolled women did not appear to have really altered metabolic values. However, our study confirms what was observed at a pilot level in terms of clinical manifestations of hyperandrogenism (Rondanelli et al., 2021) and, thanks to the results obtained in relation to menstrual and ovarian abnormalities, adds important elements that really suggest pro-fertility and pro-well-being actions on women with PCOS supplemented with berberine. In July 2023, when we were engaged in drafting the paper, two women of the berberine group became pregnant. In September 2023, during the review process of the paper, another woman of the berberine-treated group became pregnant. Bearing in mind that isolated cases obviously can only be simply anecdotal, it is worth emphasizing that one of these two women had been trying unsuccessfully for a pregnancy for 8 years and had been diagnosed with PCOS for at least 6 years. The second woman had been trying to become pregnant for 12 months. The third woman was trying to get pregnant since more than 2 years. Clearly, these results do not mean that berberine can be considered a tool to increase the chances of pregnancy. To date, in fact, there are no data available to confirm its harmlessness to foetal life. Nonetheless, our results globally demonstrate and underline once again the goodness of berberine as a useful nutraceutical tool.

The results we obtained with this study lead us to some considerations. Although the primary cause of PCOS can (could) be an abnormal production of androgens, its link with metabolic disorders has always been considered equally important. The anti-PCOS evidence described by various authors in relation to the use of metformin or berberine (or inositols) is a possible demonstration of this. In this study, however, our treatment with berberine did not noticeably modify the metabolic and/or the hormonal balance but instead have had important consequences on the menstrual cycle, the ovarian conditions and the dermatological manifestations. This finding has important implications regarding “the how” berberine has exerted these effects. It is therefore our intention to replicate this study, possibly improving its design, not only to confirm the pharmacological role played by berberine in women with PCOS when administered in forms with greater oral bioavailability, but also to be able to investigate more innovative fronts such as the analysis of the gut microbiota. The phytosome forms, in addition to being better absorbed, also improve the parameter of intestinal dispersion. And this could allow us to clearly visualize the impact of berberine on the microbiota of women with PCOS establishing a possible correlation between clinical recovery and microbiota structure before and after the treatment.

## Conclusion

In conclusion, our study confirmed the positive effects exerted by berberine in women with PCOS when administered at lower doses than those usually used because it is formulated in such a way as to become more absorbable after oral administration. At the same time, this study also confirms the high safety profile of this nutraceutical. A multi-centric, prospective, randomized, and controlled study currently underway in a European population will soon allow us to evaluate how widely the pharmacological properties observed here are extensible.

## Data Availability

The original contributions presented in the study are included in the article/Supplementary Material, further inquiries can be directed to the corresponding author.
