# Supplementary material for: Effect of Berberine Phytosome on reproductive, dermatologic, and metabolic characteristics in women with polycystic ovary syndrome: a controlled, randomized, multi-centric, open-label clinical trial
Source: Front Pharmacol. 2023 Nov 21;14:1269605. doi: 10.3389/fphar.2023.1269605 (PMC10703476; doi:10.3389/fphar.2023.1269605)
Supplement: Supplementary file 1 [file DataSheet1.PDF]

Supplementary File 1

Tests Name	Analyser	Assay /Methodology
CBC	Sysmex KX-21	electrical impedance detection technology.
CRP	Cobas Pure(303)	photometric assays
Liver Funtion Test	Cobas Pure(303)	photometric assays
GGT	Cobas Pure(303)	photometric assays
Lipid Profile	Cobas Pure(303)	photometric assays
AMH	Cobas Pure(303)	photometric assays
Fasting Glucose	Cobas Pure(303)	photometric assays
Free Testosterone	Snibe Maglumi X8	Chemiluminescence
FSH	Snibe Maglumi X8	Chemiluminescence
LH	Snibe Maglumi X8	Chemiluminescence
Fasting Insulin	Snibe Maglumi X8	Chemiluminescence
HbA1C	Bio-Rad (D-10)	High-performance liquid chromatography(HPLC)

Supplementary file 2

Subject	Centre	Group	Baseline. Menstrual cycle status	After 3-months. Menstrual cycle status
1	BMCH	Berberine	Abnormal - 10 days, irregular cycle	Improved - regular cycles, but flow is less
2	BMCH	Berberine	Abnormal	Improved - Regular, 30 days cycle, menstrual bleeding for 8 days taking medicine
3	BMCH	Berberine	Abnorml - Oligomenorrhea. Less bleeding and irregular periods, periods not come for 2-3 months	Abnorml - Oligomenorrhea
4	BMCH	Berberine	Abnorma l-Irregular Cycle outside the range	Improved - Periods Normal with normal flow
5	BMCH	Berberine	Abnorml - Oligomenorrhea - irregular periods, periods come after 2-3 months, less flow	Abnormal - Oligomenorrhea - During tsaking medicine while 3 monthspts periods, came only once but with normal flow
6	BMCH	Berberine	Abnormal - Out of the range with less bleeding	Improved - cycle became normal with normal flow
7	BMCH	Berberine	Abnormal - Oligomenorrhea	Improved - Regual Periods
8	BMCH	Berberine	Abnormal - Irregular Periods	Improved - Normal Periods
9	BMCH	Berberine	Abnormal - Irregular periods, 10-15 days cycle	Improved - Normal period, with normal flow (change 2-3 pads per day) for 5-6 days
10	BMCH	Berberine	Abnormal - Out side the normal range, Less bleeding	Improved - Normal range normal flow
11	ATH	Berberine	Abnormal - irregular 7-8 days/2-3 months	Improved -menstruated cycle after 40 days
12	ATH	Berberine	Abnormal - irregular 6 days/2-3 months	Improved - 5/35 days
13	ATH	Berberine	Abnormal - Irregular 4/2 months	Improved - 4-5 days/40 days
14	ATH	Berberine	Abnormal - irregular 3-5 days/4 months	Improved - 3-5 days/40 days
15	ATH	Berberine	Abnormal - Irregular 5 days/2 months	Improved - 4/40 days
16	ATH	Berberine	Abnormal - irregular 5 days/45 days	Improved - 5/32 days
17	ATH	Berberine	Abnormal - irregular 4-5 days/70-80 days	Improved - 5-6/40-50
18	ATH	Berberine	Abnormal - Irregular 4/2 months	Improved - 4-5 days/40 days
19	ATH	Berberine	Abnormal - irregular 2/45 days	Abnormal - 2 days/40 days
20	ATH	Berberine	Abnormal - irregular 4/60 days	Improved - now regular 4-5/40 days
21	ATH	Berberine	Abnormal - oligomennorrhea	Improved - now regular 2-3/40 days
22	ATH	Berberine	Abnormal - oligomennorrhea	Improved - regular 4/35 days
23	ATH	Berberine	Abnormal - irregular 4/60 days	Improved - 35 days
24	ATH	Berberine	Abnormal - irregular 4-5 days/45 days	Abnormal - 4-5/40 days
25	ATH	Berberine	Abnormal - irregular 6/60 days	Improved 5-6/35-45 days
26	ATH	Berberine	Abnormal - irregular 8-11 days/2-3 months	Improved - now regular 4/40 days
27	KEMU	Berberine	Abnormal - prolong cycle, sometimes 42 days, mostly 3-4 months	Improved - 24-36 days
28	KEMU	Berberine	Normal - 21-36 days cycle	Normal - 30-33 days cycle
29	KEMU	Berberine	Abnormal	Improved - Normal, but irregular spotting mostly
30	KEMU	Berberine	Abnormal	Improved - 32-37 days
31	KEMU	Berberine	Normal - 24-36 days	Normal - 30-35 days
32	KEMU	Berberine	Abnormal - 50-60 days	Improved
33	KEMU	Berberine	Abnormal (6-7 months)	
34	KEMU	Berberine	Abnormal	Abnormal - 40-55 days
35	KEMU	Berberine	Normal - 28-30 days	Normal - 29-32 days
36	KEMU	Berberine	Abnormal - Irregular	Abnormal - Irregular
37	KEMU	Berberine	Normal - Regular (3/30 days)	Normal cycle (28 days)
38	KEMU	Berberine	Normal	Normal
39	KEMU	Berberine	Abnormal (4-5 days/4-6 months)	Improved -Regular
40	KEMU	Berberine	Abnormal (After 2-3 months)	Improved -Regular
41	KEMU	Berberine	Normal (35-45 days)	Normal
42	LRH	Berberine	Abnormal -7/60 days	Improved - Regular 7/30 days
43	LRH	Berberine	Abnormal -7/2 months	Improved- 7/40 days
44	LRH	Berberine	Abnormal	Improved - Regular 7/30 days
45	LRH	Berberine	Abnormal-7/11-24 months	Improved to Normal
46	LRH	Berberine	Abnormal -5/60 days	Abnormal - 5/30-70 days
47	LRH	Berberine	Abnormal-7/50 days	Improved - Regular 7/30 days
48	LRH	Berberine	Abnormal-Irregular	Abnorma- Irregular 10/50 days
49	LRH	Berberine	Abnormal -7/60-90 days	Improved - Normal
50	LRH	Berberine	Abnormal - Irregular	Abnormal - 6/65 days
51	LRH	Berberine	Abnormal - 3-7/30-60 days	Improved - Regular 7/30 days
52	KTH	Control	Abnorml - Oligomenorrhea	Abnorml - Oligomenorrhea
53	KTH	Control	Abnorml - Oligomenorrhea (3-5 days/3-5 months)	Abnorml - Oligomenorrhea
54	KTH	Control	Abnormal - Oligomenorrheic. Cycle 4-5 d/50-60 days	Abnormal - Erratic. 4 times in the last 3 months
55	KTH	Control	Abnorml (5/40-45)	Abnormal - 5/40-45
56	KTH	Control	Abnorml - Oligomenorrhea	Abnormal - Amenorrheic since 2 months
57	KTH	Control	Abnorml - Oligomenorrhea	Abnorml - Oligomenorrhea
58	KTH	Control	Abnorml - Oligomenorrhea, comes every 6-8 months, scanty	Abnormal - Erratic cycle. Bled 4 times in the last 3 months
59	KTH	Control	Abnormal - Erratic. Twice in a month , continues for >10 days.	Abnormal - Irregular. 3 times in the last 1 month.
60	KTH	Control	Abnorml - Oligomenorrhea. Comes after 4-5 months.	Abnorml - Oligomenorrhea.
61	BMCH	Control	Abnormal - ireegular menstrual cycle.periods lasts max 2 days and have painful periods	Abnormal - periods are not regular
62	BMCH	Control	Abnormal - Oligomenorrhea, get periods after 2 to 3 months	Abnormal - Oligomenorrhea, get periods after 2 to 3 months
63	BMCH	Control	Abnorml - Oligomenorrhea - only get period for 2-3 times totally	Abnorml - Oligomenorrhea
64	BMCH	Control	Abnormal - Short Menstrual cycle	Abnormal - very irregular periods
65	ATH	Control	Abnormal - irregular 5/60-90 days	Abnormal - 5/60 days
66	ATH	Control	Abnormal - irregular 3/45 days	Abnormal - irregular
67	ATH	Control	Abnormal - hypooligomennorrhea	Improved - 3-5 days/40 days
68	ATH	Control	Abnormal - irregular	Abnormal - menstruated only once for 5 days in these 3 months
69	ATH	Control	Abnormal - irregular 2/120 days	Abnormal - 2/58 days
70	ATH	Control	Abnormal - irregular 2/42-52 days	Abnormal - 2/40 days
71	ATH	Control	Abnormal - irregular 7-11 days/4-6 months	Anormal - Did not mentsruate whithin these 3 months
72	ATH	Control	Abnormal - irregular 4/90 days	Abnormal - 4/80 days
73	ATH	Control	Abnormal - irregular 4/54 days	Improved - only once for 4 days in the 20 days
74	ATH	Control	Abnormal - irregular 4/60 days	Improved - Regular 5/40 days
75	ATH	Control	Abnormal - irregular 4/60 days	Improved 4/40 days
76	ATH	Control	Abnormal - irregular 2-3 days/60 days	Abnormal - 5-6/40-60 days
77	ATH	Control	Abnormal - irregular 8-9 days/4-6 months	Abnormal - 7-8/4-6 months
78	ATH	Control	Abnormal - irregular 7-11 days/60-70 days	Improved - irregular 3-5/40 days
79	ATH	Control	Abnormal - irregular 3/46 days	Abnormal - 3/35 days
80	ATH	Control	Abnormal - irregular, scanty 6-11 days/4-6 months	Abnormal - now menstruated after 50 days for 8 days
81	KEMU	Control	Abnormal	Abnormal
82	KEMU	Control	Abnormal	Abnormal - 40-45 days
83	KEMU	Control	Normal	Normal - 30-34 days
84	KEMU	Control	Abnormal	Improved sligthly, 35-50 days
85	KEMU	Control	Abnormal - Irregular	Abnormal - Irregular
86	KEMU	Control	Abnormal - Irregular	Abnormal - 35-45 days
87	KEMU	Control	Normal - Regular (35 days)	Normal - 35-39 days
88	KEMU	Control	Abnormal - Irregular	Abnormal - 30-42 days
89	KEMU	Control	Abnormal - Irregular	Abnormal - Irregular
90	KEMU	Control	Abnorml - Oligomenorrhea - No mentsrual cycle for 1 year	Improved - Regular (4 days/28 days)
91	KEMU	Control	Normal - Regular (32 days)	Normal - Regular (45 days)
92	KEMU	Control	Abnormal	Abnormal
93	KEMU	Control	Abnormal - Irregular (2-3 months)	Improved - Normal (30 days)
94	KEMU	Control	Abnormal	Abnormal
95	KEMU	Control	Normal	Abnormal
96	KEMU	Control	Abnormal	Abnormal
97	LRH	Control	Abnormal -Irregular	Irregular 7/15 days
98	LRH	Control	Abnormal - 7-10/60-75 days	Abnormal - 7/45 days
99	LRH	Control	Abnormal - Irregular	Abnormal - 7/50-65 days
100	LRH	Control	Abnormal - Irregular	Abnormal -Irregular
101	LRH	Control	Abnormal - 7/60 days	Abnormal - 7/35-45 days
102	LRH	Control	Abnormal - Irregular	Abnormal - 7/40-45 days
103	LRH	Control	Abnormal - Irregular	Abnormal -Irregular
104	LRH	Control	Abnormal - Normal	Improved to normal 7/30 days
105	LRH	Control	Abnormal - 5/3-4 months	Abnormal - 3/60-90 days
106	LRH	Control	Normal	Normal

Supplementary file 3

Categorical

Repeated Measures(Baseline. Menstrual cycle status, After 3-months. Menstrual cycle status) By Group

Freq<br>Share<br>Comparisons		Response: Baseline. Menstrual cycle status — Group: Berberine<br>A	Control<br>B	Response: After 3-months. Menstrual cycle status — Group: Berberine<br>C	Control<br>D
Response	Normal	6<br>11.8%<br>*	5<br>9.1%<br>*	6<br>12.0%<br>*	4<br>7.3%<br>*
	Abnormal	45<br>88.2%<br>*	50<br>90.9%<br>*	9<br>18.0%<br>*	41<br>74.5%<br>C*
	Improved	0<br>0.0%<br>*	0<br>0.0%<br>*	35<br>70.0%<br>D*	9<br>16.4%<br>*
	Irregular	0<br>0.0%<br>*	0<br>0.0%<br>*	0<br>0.0%<br>*	1<br>1.8%<br>*
	Total Responses	51	55	50	55
	Compare	*	*	*	C*

Default Comparison Groups: A/B,C/D

Shows letter of the category it is significantly different from at the higher share level

* Base count warning	100	Uppercase Alpha Level	0.05
** Base count minimum	30	Lowercase Alpha Level	0.1

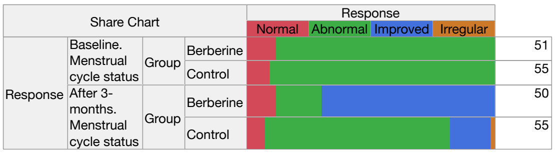

Compare Each Sample

Letter comparisons use Pearson Chisq

Group, Baseline. Menstrual cycle status

LR PValues — LR Chi-square p-value on pairs

	A	B
A	1.0000	0.6520
B	0.6520	1.0000

Pearson PValues — Pearson Chi-square p-value on pairs

	A	B
A	1.0000	0.6520
B	0.6520	1.0000

Group, After 3-months. Menstrual cycle status

LR Chi-square p-value on pairs

	C	D
C	1.0000	<.0001
D	<.0001	1.0000

Pearson Chi-square p-value on pairs

	C	D
C	1.0000	<.0001
D	<.0001	1.0000

Compare Each Cell - Details

Letter comparisons use Fisher's Exact Test

Group, Baseline. Menstrual cycle status — LR Pairs

		AB	BB
Normal	1.0000	0.6520	1.0000
Abnormal	1.0000	0.6520	1.0000
Improved	1.0000	.	1.0000
Irregular	1.0000	.	1.0000

Group, After 3-months. Menstrual cycle status — LR Pairs

		CD	DD
Normal	1.0000	0.4093	1.0000
Abnormal	1.0000	0.0000	1.0000
Improved	1.0000	0.0000	1.0000
Irregular	1.0000	0.2538	1.0000

Pearson Pairs (Baseline)

		AB	BB
Normal	1.0000	0.6520	1.0000
Abnormal	1.0000	0.6520	1.0000
Improved	1.0000	.	1.0000
Irregular	1.0000	.	1.0000

Pearson Pairs (After 3-months)

		CD	DD
Normal	1.0000	0.4099	1.0000
Abnormal	1.0000	0.0000	1.0000
Improved	1.0000	0.0000	1.0000
Irregular	1.0000	0.3380	1.0000

Fisher Exact Pairs (Baseline)

		AB	BB
Normal	1.0000	0.7550	1.0000
Abnormal	1.0000	0.7550	1.0000
Improved	1.0000	1.0000	1.0000
Irregular	1.0000	1.0000	1.0000

Fisher Exact Pairs (After 3-months)

		CD	DD
Normal	1.0000	0.5131	1.0000
Abnormal	1.0000	0.0000	1.0000
Improved	1.0000	0.0000	1.0000
Irregular	1.0000	1.0000	1.0000

Transition Report

Group: Berberine — From: Baseline. Menstrual cycle status — to: After 3-months. Menstrual cycle status

Transition Counts

	Normal	Abnormal	Improved	Irregular
Normal	6	0	0	0
Abnormal	0	9	35	0
Improved	0	0	0	0
Irregular	0	0	0	0

Transition Rates

	Normal	Abnormal	Improved	Irregular	After 3-months. Menstrual cycle status
Normal	1.0000	0.0000	0.0000	0.0000	
Abnormal	0.0000	0.2045	0.7955	0.0000	
Improved	.	.	.	.	
Irregular	.	.	.	.	

Group: Control — From: Baseline. Menstrual cycle status — to: After 3-months. Menstrual cycle status

Transition Counts

	Normal	Abnormal	Improved	Irregular
Normal	4	1	0	0
Abnormal	0	40	9	1
Improved	0	0	0	0
Irregular	0	0	0	0

Transition Rates

	Normal	Abnormal	Improved	Irregular	After 3-months. Menstrual cycle status
Normal	0.8000	0.2000	0.0000	0.0000	
Abnormal	0.0000	0.8000	0.1800	0.0200	
Improved	.	.	.	.	
Irregular	.	.	.	.	

Group: All — From: Baseline. Menstrual cycle status — to: After 3-months. Menstrual cycle status

Transition Counts

	Normal	Abnormal	Improved	Irregular
Normal	10	1	0	0
Abnormal	0	49	44	1
Improved	0	0	0	0
Irregular	0	0	0	0

Transition Rates

	Normal	Abnormal	Improved	Irregular	After 3-months. Menstrual cycle status
Normal	0.9091	0.0909	0.0000	0.0000	
Abnormal	0.0000	0.5213	0.4681	0.0106	
Improved	.	.	.	.	
Irregular	.	.	.	.	

Supplementary file 4

Subject	Group	Baseline. Pelvic ultrasound Findings	After 3-months. Pelvic ultrasound Findings
1	Berberine	PCOS- bilateral with thick central echogenic strom	PCOS (Bilateral)
2	Berberine	PCOS (Bilateral)	Improvement: Left ovary showing improvement with 1 dominating follicle.
3	Berberine	Normal ovary	Normal ovary
4	Berberine	PCOS (Bilateral)	PCOS (Bilateral)
5	Berberine	PCOS (Bilateral)	PCOS (Bilateral)
6	Berberine	PCOS (Bilateral)	Improvement: in the right ovary showing 1 dominant follice.
7	Berberine	Normal ovary	Normal ovary
8	Berberine	PCOS (Bilateral)	PCOS (Bilateral)
9	Berberine	PCOS (Bilateral)	PCOS (Bilateral)
10	Berberine	PCOS (Bilateral)	Improvement: Normal Ovary
11	Berberine	PCOS (Bilateral), thick storma	Improvement (sligth): stroma less thick
12	Berberine	PCOS (Bilateral), thick storma	Improvement: Adnexa clears, No PCOS
13	Berberine	PCOS (Bilateral)	Improvement
14	Berberine	PCOS (Bilateral)	Improvement: Right ovary has developing follicles Left ovary PCOS
15	Berberine	PCOS (Bilateral), thick storma	Improvement: Adnexa clears, No PCOS
16	Berberine	PCOS (Bilateral)	Improvement: Left ovary has one large developing follicles. Right ovary PCOS
17	Berberine	PCOS (Bilateral)	PCOS (Bilateral), thick storma
18	Berberine	PCOS (Bilateral)	Improvement: Right ovary has developing follicles showing. Left ovary PCOS
19	Berberine	PCOS (Bilateral)	Improvement : Two enlarging follicles 1.3 cm in right ovary, and 1.7 cm in left ovary.
20	Berberine	PCOS (Bilateral)	PCOS (Bilateral)
21	Berberine	PCOS (Bilateral)	PCOS (Bilateral)
22	Berberine	PCOS (Bilateral)	Improvement in Right ovary. Left ovary PCOS
23	Berberine	PCOS (Bilateral)	Improvement: Left ovary showing two large follicles 1.4 cm, 1.1 cm. Right ovary PCOS
24	Berberine	PCOS (Bilateral)	Improvement
25	Berberine	PCOS (Bilateral)	Improvement
26	Berberine	PCOS (Bilateral), thick storma	Improvement: Adnexa clears, No PCOS
27	Berberine	PCOS positive	PCOS positive
28	Berberine	PCOS positive	Improvement: PCOS cleared
29	Berberine	PCOS positive	Improvement: PCOS cleared
30	Berberine	PCOS positive	Improvement
31	Berberine	PCOS positive	PCOS positive
32	Berberine	PCOS positive	PCOS positive
33	Berberine	PCOS positive	Improvement: PCOS cleared
34	Berberine	PCOS positive	Improvement
35	Berberine	PCOS positive	PCOS (Bilateral)
36	Berberine	PCOS positive. Uterus A/V, 6mm thick endometrium	Improvement
37	Berberine	PCOS positive	Improvement
38	Berberine	Normal Ovary	Improvement: PCOS cleared
39	Berberine	PCOS (Bilateral)	Improvement: Left ovary with one dominant follicle.
40	Berberine	PCOS positive. A/V uterus	PCOS positive. A/V uterus.B/L Adenexa.
41	Berberine	PCOS (Bilateral)	PCOS (Bilateral)
42	Berberine	PCOS Positive	Improvement: PCOS cleared
43	Berberine	PCOS (Bilateral)	Improvement:
44	Berberine	Normal ovary	Normal ovary
45	Berberine	Normal ovary	Normal ovary
46	Berberine	PCOS Positive	Improvement: PCOS cleared
47	Berberine	PCOS Positive	Improvement: PCOS cleared
48	Berberine	PCOS Positive	Improvement: PCOS cleared
49	Berberine	PCOS Positive	Improvement: PCOS cleared
50	Berberine	PCOS Positive	Improvement: Normal ovary
51	Berberine	PCOS (Bilateral)	Improvement: PCOS cleared
52	Control	PCOS (Bilateral)	PCOS (Bilateral)
53	Control	PCOS	PCOS
54	Control	PCOS	PCOS
55	Control	PCOS	PCOS
56	Control	PCOS	PCOS
57	Control	PCOS	PCOS
58	Control	PCOS	PCOS (left ovary)
59	Control	PCOS (Bilateral)	PCOS (Bilateral)
60	Control	PCOS	PCOS
61	Control	PCOS (Bilateral)	Improvement: Normal Ovary
62	Control	PCOS (Bilateral)	PCOS (Bilateral)
63	Control	PCOS (Bilateral)	Improvement: Left ovary showing with 1 dominating follicle.
64	Control	PCOS (Bilateral)	PCOS (Bilateral)
65	Control	PCOS (Bilateral)	PCOS (Bilateral)
66	Control	PCOS (Bilateral)	PCOS (Bilateral)
67	Control	PCOS (Bilateral)	PCOS (Bilateral), thick storma
68	Control	PCOS (Bilateral)	PCOS (Bilateral)
69	Control	PCOS (Bilateral)	Improvement: Right ovary showing developing follicles. Left still PCOS
70	Control	PCOS (Bilateral)	PCOS (Bilateral)
71	Control	PCOS (Bilateral)	PCOS (Bilateral), thick storma
72	Control	PCOS (Bilateral)	PCOS (Bilateral)
73	Control	PCOS (Bilateral)	PCOS (Bilateral)
74	Control	PCOS left ovary. Right ovary Hemorrhagic ovarian cyst.	Improvement: Left ovary PCOS. Right ovary normal looking
75	Control	PCOS (Bilateral)	PCOS (Bilateral), thick storma
76	Control	PCOS (Bilateral)	PCOS (Bilateral)
77	Control	PCOS (Bilateral)	PCOS (Bilateral)
78	Control	PCOS (Bilateral)	PCOS (Bilateral), thick storma
79	Control	PCOS (Bilateral)	Improvement
80	Control	PCOS (Bilateral)	PCOS (Bilateral), thick storma
81	Control	PCOS positive	PCOS positive
82	Control	PCOS positive	PCOS positive
83	Control	PCOS positive	Improvement: PCOS cleared
84	Control	PCOS positive	PCOS positive
85	Control	PCOS positive	PCOS positive
86	Control	PCOS positive	PCOS positive
87	Control	PCOS positive	PCOS positive
88	Control	PCOS positive	Improvement
89	Control	PCOS (Bilateral)	PCOS positive
90	Control	PCOS positive. A/V uterus, thick endometrius	Improvement
91	Control	PCOS (Bilateral)	PCOS (Bilateral)
92	Control	PCOS (Bilateral)	PCOS (Bilateral)
93	Control	PCOS (Bilateral)	PCOS (Bilateral)
94	Control	PCOS (Bilateral)	PCOS (Bilateral)
95	Control	PCOS (Bilateral)	PCOS (Bilateral)
96	Control	PCOS (Bilateral)	PCOS (Bilateral)
97	Control	PCOS (Bilateral)	PCOS Positive
98	Control	PCOS Positive	Improvement: Normal ovary
99	Control	PCOS Positive	Improvement: PCOS cleared
100	Control	PCOS Positive	Improvement: PCOS cleared
101	Control	PCOS Positive	Improvement: PCOS cleared
102	Control	PCOS (Bilateral)	PCOS Positive
103	Control	Normal ovary	Normal ovary
104	Control	PCOS Positive	Improvement: Normal ovary
105	Control	Normal ovary	Improvement: Normal ovary
106	Control	PCOS Positive	PCOS (Bilateral)

Supplementary file 5

Categorical

Repeated Measures(Baseline. Pelvic ultrasound Findings, After 3-months. Pelvic ultrasound Findings) By Group

Freq / Share / Comparisons		Response: Baseline. Pelvic ultrasound Findings – Group: Berberine (A)	Response: Baseline. Pelvic ultrasound Findings – Group: Control (B)	Response: After 3-months. Pelvic ultrasound Findings – Group: Berberine (C)	Response: After 3-months. Pelvic ultrasound Findings – Group: Control (D)
Response	Normal	5	2	4	1
		9.8%	3.6%	7.8%	1.8%
		*	*	*	*
	PCOS	46	53	14	40
		90.2%	96.4%	27.5%	72.7%
		*	*	*	C*
	Improvement	0	0	33	14
		0.0%	0.0%	64.7%	25.5%
		*	*	D*	*
	Total Responses	51	55	51	55
	Compare	*	*	*	C*

Default Comparison Groups: A/B,C/D

Shows letter of the category it is significantly different from at the higher share level

* Base count warning	100	Uppercase Alpha Level	0.05
** Base count minimum	30	Lowercase Alpha Level	0.1

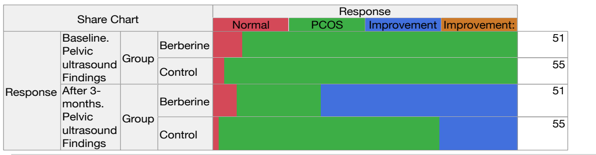

Compare Each Sample

Letter comparisons use Pearson Chisq

Group, Baseline. Pelvic ultrasound Findings

LR PValues — LR Chi-square p-value on pairs

	A	B
A	1.0000	0.1959
B	0.1959	1.0000

Pearson PValues — Pearson Chi-square p-value on pairs

	A	B
A	1.0000	0.2014
B	0.2014	1.0000

Group, After 3-months. Pelvic ultrasound Findings

LR Chi-square p-value on pairs

	C	D
C	1.0000	<.0001
D	<.0001	1.0000

Pearson Chi-square p-value on pairs

	C	D
C	1.0000	<.0001
D	<.0001	1.0000

Compare Each Cell - Details

Letter comparisons use Fisher's Exact Test

Group, Baseline. Pelvic ultrasound Findings

LR Pairs

		AB	BB
Normal	1.0000	0.1959	1.0000
PCOS	1.0000	0.1959	1.0000
Improvement	1.0000	.	1.0000
Improvement:	1.0000	.	1.0000

Pearson Pairs

		AB	BB
Normal	1.0000	0.2014	1.0000
PCOS	1.0000	0.2014	1.0000
Improvement	1.0000	.	1.0000
Improvement:	1.0000	.	1.0000

Fisher Exact Pairs

		AB	BB
Normal	1.0000	0.2575	1.0000
PCOS	1.0000	0.2575	1.0000
Improvement	1.0000	1.0000	1.0000
Improvement:	1.0000	1.0000	1.0000

Group, After 3-months. Pelvic ultrasound Findings

LR Pairs

		CD	DD
Normal	1.0000	0.1326	1.0000
PCOS	1.0000	0.0000	1.0000
Improvement	1.0000	0.0000	1.0000
Improvement:	1.0000	.	1.0000

Pearson Pairs

		CD	DD
Normal	1.0000	0.1438	1.0000
PCOS	1.0000	0.0000	1.0000
Improvement	1.0000	0.0000	1.0000
Improvement:	1.0000	.	1.0000

Fisher Exact Pairs

		CD	DD
Normal	1.0000	0.1931	1.0000
PCOS	1.0000	0.0000	1.0000
Improvement	1.0000	0.0001	1.0000
Improvement:	1.0000	1.0000	1.0000

Transition Report

Group: Berberine — From: Baseline. Pelvic ultrasound Findings — to: After 3-months. Pelvic ultrasound Findings

Transition Counts

	Normal	PCOS	Improvement	Improvement:
Normal	4	0	1	0
PCOS	0	14	32	0
Improvement	0	0	0	0
Improvement:	0	0	0	0

Transition Rates

	Normal	PCOS	Improvement	Improvement:	After 3-months. Pelvic ultrasound Findings
Normal	0.8000	0.0000	0.2000	0.0000	
PCOS	0.0000	0.3043	0.6957	0.0000	
Improvement	.	.	.	.	
Improvement:	.	.	.	.	

Group: Control — From: Baseline. Pelvic ultrasound Findings — to: After 3-months. Pelvic ultrasound Findings

Transition Counts

	Normal	PCOS	Improvement	Improvement:
Normal	1	0	1	0
PCOS	0	40	13	0
Improvement	0	0	0	0
Improvement:	0	0	0	0

Transition Rates

	Normal	PCOS	Improvement	Improvement:	After 3-months. Pelvic ultrasound Findings
Normal	0.5000	0.0000	0.5000	0.0000	
PCOS	0.0000	0.7547	0.2453	0.0000	
Improvement	.	.	.	.	
Improvement:	.	.	.	.	

Group: All — From: Baseline. Pelvic ultrasound Findings — to: After 3-months. Pelvic ultrasound Findings

Transition Counts

	Normal	PCOS	Improvement	Improvement:
Normal	5	0	2	0
PCOS	0	54	45	0
Improvement	0	0	0	0
Improvement:	0	0	0	0

Transition Rates

	Normal	PCOS	Improvement	Improvement:	After 3-months. Pelvic ultrasound Findings
Normal	0.7143	0.0000	0.2857	0.0000	
PCOS	0.0000	0.5455	0.4545	0.0000	
Improvement	.	.	.	.	
Improvement:	.	.	.	.	

Supplementary file 6

Subjects	Group	Baseline. Presence of Acne	After 3-months. Presence of Acne
1	Berberine	Yes	Improved
2	Berberine	Yes	Improved
3	Berberine	No	No
4	Berberine	Yes	Improved
5	Berberine	Yes	improved
6	Berberine	Yes	Improved
7	Berberine	No	Yes
8	Berberine	Yes	Improved
9	Berberine	Yes	No
10	Berberine	No	No
11	Berberine	No	No
12	Berberine	No	No
13	Berberine	No	No
14	Berberine	Yes	Yes
15	Berberine	Yes	Improved symptoms
16	Berberine	Yes	Improved symptoms
17	Berberine	No	No
18	Berberine	Yes	Yes
19	Berberine	No	No
20	Berberine	No	No
21	Berberine	No	No
22	Berberine	No	No
23	Berberine	Yes	Improved symptoms
24	Berberine	No	No
25	Berberine	No	No
26	Berberine	Yes	Yes
27	Berberine	Yes	Yes
28	Berberine	Yes	No
29	Berberine	Yes	Improved
30	Berberine	No	No
31	Berberine	Yes	Yes
32	Berberine	Yes	Improved
33	Berberine	Yes	Yes
34	Berberine	Yes	Improved
35	Berberine	No	No
36	Berberine	No	No
37	Berberine	No	No
38	Berberine	Yes	Yes
39	Berberine	Yes	No
40	Berberine	No	No
41	Berberine	No	No
42	Berberine	Yes	Yes
43	Berberine	No	No
44	Berberine	Yes	Yes
45	Berberine	Yes	Yes
46	Berberine	Yes	No
47	Berberine	Yes	Yes
48	Berberine	Yes	Yes
49	Berberine	Yes	No
50	Berberine	No	no
51	Berberine	No	No
52	Control	No	No
53	Control	No	No
54	Control	Yes	Yes
55	Control	Yes	Yes
56	Control	Yes	No
57	Control	Yes	Yes
58	Control	Yes	Improved
59	Control	Yes	Improved
60	Control	Yes	Yes
61	Control	No	No
62	Control	No	No
63	Control	Yes	Yes
64	Control	Yes	Yes
65	Control	No	No
66	Control	Yes	Yes
67	Control	No	No
68	Control	Yes	Yes
69	Control	Yes	Yes
70	Control	No	No
71	Control	No	No
72	Control	No	yes, no improvement
73	Control	Yes	Yes
74	Control	Yes	Yes
75	Control	No	No
76	Control	Yes	Improved symptoms
77	Control	Yes	Yes
78	Control	Yes	Yes
79	Control	yes	No
80	Control	Yes	No
81	Control	No	Yes
82	Control	Yes	Yes
83	Control	Yes	Improved
84	Control	Yes	Improved
85	Control	No	Mild
86	Control	No	No
87	Control	No	No
88	Control	No	No
89	Control	No	No
90	Control	No	No
91	Control	No	No
92	Control	No	No
93	Control	Yes	No
94	Control	Yes	Yes
95	Control	Yes	Yes
96	Control	Yes	Yes
97	Control	No	No
98	Control	No	no
99	Control	No	No
100	Control	Yes	Yes
101	Control	No	No
102	Control	No	No
103	Control	No	No
104	Control	No	no
105	Control	Yes	no
106	Control	No	No

Supplementary file 7

Categorical

Repeated Measures(Baseline. Presence of Acne, After 3-months. Presence of Acne) By Group

Freq / Share / Comparisons		Baseline. Presence of Acne: Group Berberine A	Baseline. Presence of Acne: Group Control B	After 3-months. Presence of Acne: Group Berberine C	After 3-months. Presence of Acne: Group Control D
Response	No	22<br>43.1%<br>*	27<br>49.1%<br>*	26<br>51.0%<br>*	30<br>54.5%<br>*
	Mild	0<br>0.0%<br>*	0<br>0.0%<br>*	0<br>0.0%<br>*	1<br>1.8%<br>*
	Improved	0<br>0.0%<br>*	0<br>0.0%<br>*	12<br>23.5%<br>d*	5<br>9.1%<br>*
	Yes	29<br>56.9%<br>*	28<br>50.9%<br>*	13<br>25.5%<br>*	19<br>34.5%<br>*
	Total Responses	51	55	51	55
	Compare	*	*	*	*

Default Comparison Groups: A/B,C/D

Shows letter of the category it is significantly different from at the higher share level

* Base count warning	100	Uppercase Alpha Level	0.05
** Base count minimum	30	Lowercase Alpha Level	0.1

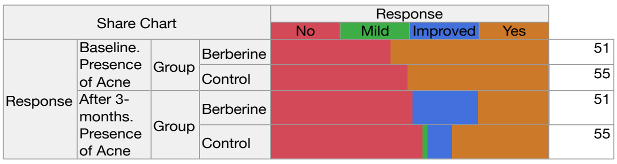

Compare Each Sample

Letter comparisons use Pearson Chisq

LR PValues / Pearson PValues

Group, Baseline. Presence of Acne

LR Chi-square p-value on pairs

	A	B
A	1.0000	0.5389
B	0.5389	1.0000

Pearson Chi-square p-value on pairs

	A	B
A	1.0000	0.5390
B	0.5390	1.0000

Group, After 3-months. Presence of Acne

LR Chi-square p-value on pairs

	C	D
C	1.0000	0.1315
D	0.1315	1.0000

Pearson Chi-square p-value on pairs

	C	D
C	1.0000	0.1612
D	0.1612	1.0000

Compare Each Cell - Details

Letter comparisons use Fisher's Exact Test

Group, Baseline. Presence of Acne — LR Pairs

		AB	BB
No	1.0000	0.5389	1.0000
Mild	1.0000	.	1.0000
Improved	1.0000	.	1.0000
Yes	1.0000	0.5389	1.0000

Group, After 3-months. Presence of Acne — LR Pairs

		CD	DD
No	1.0000	0.7133	1.0000
Mild	1.0000	0.2504	1.0000
Improved	1.0000	0.0409	1.0000
Yes	1.0000	0.3090	1.0000

Group, Baseline. Presence of Acne — Pearson Pairs

		AB	BB
No	1.0000	0.5390	1.0000
Mild	1.0000	.	1.0000
Improved	1.0000	.	1.0000
Yes	1.0000	0.5390	1.0000

Group, After 3-months. Presence of Acne — Pearson Pairs

		CD	DD
No	1.0000	0.7133	1.0000
Mild	1.0000	0.3333	1.0000
Improved	1.0000	0.0430	1.0000
Yes	1.0000	0.3103	1.0000

Group, Baseline. Presence of Acne — Fisher Exact Pairs

		AB	BB
No	1.0000	0.5642	1.0000
Mild	1.0000	1.0000	1.0000
Improved	1.0000	1.0000	1.0000
Yes	1.0000	0.5642	1.0000

Group, After 3-months. Presence of Acne — Fisher Exact Pairs

		CD	DD
No	1.0000	0.8458	1.0000
Mild	1.0000	1.0000	1.0000
Improved	1.0000	0.0627	1.0000
Yes	1.0000	0.3978	1.0000

Transition Report

Group: Berberine — From: Baseline. Presence of Acne — to: After 3-months. Presence of Acne

Transition Counts

	No	Mild	Improved	Yes
No	21	0	0	1
Mild	0	0	0	0
Improved	0	0	0	0
Yes	5	0	12	12

Transition Rates

	No	Mild	Improved	Yes	After 3-months. Presence of Acne
No	0.9545	0.0000	0.0000	0.0455	
Mild	.	.	.	.	
Improved	.	.	.	.	
Yes	0.1724	0.0000	0.4138	0.4138	

Group: Control — From: Baseline. Presence of Acne — to: After 3-months. Presence of Acne

Transition Counts

	No	Mild	Improved	Yes
No	25	1	0	1
Mild	0	0	0	0
Improved	0	0	0	0
Yes	5	0	5	18

Transition Rates

	No	Mild	Improved	Yes	After 3-months. Presence of Acne
No	0.9259	0.0370	0.0000	0.0370	
Mild	.	.	.	.	
Improved	.	.	.	.	
Yes	0.1786	0.0000	0.1786	0.6429	

Group: All — From: Baseline. Presence of Acne — to: After 3-months. Presence of Acne

Transition Counts

	No	Mild	Improved	Yes
No	46	1	0	2
Mild	0	0	0	0
Improved	0	0	0	0
Yes	10	0	17	30

Transition Rates

	No	Mild	Improved	Yes	After 3-months. Presence of Acne
No	0.9388	0.0204	0.0000	0.0408	
Mild	.	.	.	.	
Improved	.	.	.	.	
Yes	0.1754	0.0000	0.2982	0.5263	

Supplementary File 8

Berberine Phytosome, technology and PK in humans

(data from: Petrangolini G, Corti F, Ronchi M, Arnoldi L, Allegrini P, Riva A. Development of an Innovative Berberine Food-Grade Formulation with an Ameliorated Absorption: In Vitro Evidence Confirmed by Healthy Human Volunteers Pharmacokinetic Study. Evid Based Complement Alternat Med. 2021 Nov 27;2021:7563889. doi: 10.1155/2021/7563889. PMID: 34904017; PMCID: PMC8665891).

Berberine Phytosome® (BBR-PP, berberine phospholipids/PRO, Patent Application WO2019/150225) is a solid dispersion containing berberine extract in a rational combination with sunflower lecithin, pea protein (NUTRALYS® S85F, supplied by Roquette Freres, Lestrem, France), and grape seed extract. Berberine extract is obtained by aqueous extraction from the roots of Berberis aristata. Procedures involved grinding the roots, extraction in an aqueous medium, precipitation, and drying. The Vitis vinifera grape seed extract is a proprietary oligomeric proanthocyanidin (OPC) standardized extract (Enovita®) made exclusively with grape seeds from white wine production. Using only water as extraction solvent, grape seed extract is standardized to provide 95.0% of OPCs by spectrophotometry and a relatively low amount of flavane monomers (5.0–15.0% catechin and epicatechin, by HPLC). Finally, food-grade hydroxypropylcellulose (Klucel) and amorphous silica (SYLOID) have been also added to improve the physical and technological properties of the Phytosome® and to facilitate its incorporation in different dosage forms. BBR-PP is standardized to contain 28–34% of berberine (by HPLC). BBR-PP is obtained by the solvent evaporation method which involves the co-solubilisation and co-dispersion of berberine extract and of the other components, with the exception of silicon dioxide, in an organic solvent. The organic solvent is then removed under reduced pressure and silicon dioxide is added to the dry powder to improve the flowability during the final calibration step to obtain a suitable granulometry.

BBR-PP was orally administered to healthy volunteers, demonstrates a highly significant increase in plasma berberine concentrations versus the unformulated berberine. A 4-fold to 6-fold increase in AUC (calculated on total berberine) with respect to berberine chloride treated group is observed after BBR-PP administration. That increase is up to tenfold by considering the effective berberine content in the tested tablets (452 mg for berberine chloride and 188 mg for BBR-PP). At 24 hours after treatment with BBR-PP, berberine plasma levels are quite detectable and allow the assumption that, with repeated administration, it is possible to achieve the steady state.
